# Hyperdilute Radiesse Preserves Facial Volume in Glucagon-Like Peptide-1 Receptor Agonist Users Undergoing Rapid Weight Loss

**DOI:** 10.1093/asjof/ojaf088

**Published:** 2025-10-21

**Authors:** K Kay Durairaj, Alec D McCarthy, Monalea Yambao, Jacob Linnemann-Heath

## Abstract

Biostimulators like hyperdiluted calcium hydroxylapatite-carboxymethylcellulose (CaHA-CMC; Radiesse, Merz North America, Franksville, WI) are effective in promoting neocollagenesis and restoring facial volume. The emergence of glucagon-like peptide-1 (GLP-1) receptor agonists (GLP1RAs) for weight loss has introduced a subset of patients experiencing rapid weight loss with associated facial-volume changes, colloquially known as “Ozempic Face” (Novo Nordisk, Bagsvaerd, Denmark). This case series describes 4 patients on GLP1RAs who, despite rapid weight loss, maintained facial volume following treatment with hypderilute CaHA-CMC in the lower face. Four patients undergoing rapid weight loss with GLP1RA were retrospectively evaluated following hyperdilute (1:3) CaHA-CMC treatment in the lower face. Patient demographics, BMI, average weight loss, and follow-up assessments were reviewed. Responses were evaluated using standardized photographs, the Global Aesthetic Improvement Scale (GAIS), and patient satisfaction surveys. All 4 GLP1RA users completed both treatment sessions and 6 months follow-up. They lost an average of 24.3 ± 10.4 lb, equivalent to −9.2 ± 4.8% of baseline body weight, yet objective facial metrics remained stable or improved. Average cheek volume increased 9.8%, jowl volume declined 55.8%, nasolabial-fold depth decreased 46.2%, and marionette-line depth decreased 20.6%. At 6 months, investigator GAIS rated every patient “improved” or “much improved,” and 100% of patients reported being “satisfied” or “very satisfied.” Hyperdilute CaHA-CMC may offer benefits in preserving facial volume for patients undergoing weight loss with GLP-1 agonists. Further studies are warranted to elucidate the mechanisms and optimize treatment protocols for this population.

**Level of Evidence: 4 (Therapeutic)**

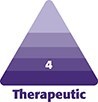

Biostimulators have become integral to aesthetic medicine for their ability to gradually volumize tissues through extracellular matrix (ECM) synthesis.^1^ Calcium hydroxylapatite-carboxymethylcellulose (CaHA-CMC; Radiesse, North America, Franksville, WI) is routinely diluted with an aqueous solution and used as a biostimulator to address facial-volume loss and skin laxity.^2,3^ When hyperdiluted, the direct filling properties of CaHA-CMC are almost entirely eliminated, and thus the primary mechanism resulting in aesthetic correction is ECM protein synthesis, building improved collagen scaffolding, and improving skin foundation.^4,5^

The explosive rise in the use of glucagon-like peptide-1 (GLP-1) receptor agonists (GLP1RAs) for weight management has introduced a unique challenge in aesthetic medicine.^6^ Rapid weight loss induced by these medications often results in noticeable facial-volume loss, colloquially referred to as “Ozempic Face” (semaglutide; Novo Nordisk, Bagsvaerd, Denmark).^7^ Rapid weight loss can lead to noticeable reductions in facial volume because of decreases in subcutaneous fat and diminished support for overlying skin. Because adipose tissue in the face is lost, the cheeks may appear hollow and facial contours sharper, which can contribute to a more gaunt, aged appearance. Although these changes are not unique to GLP1RA use and can occur with any rapid weight reduction, the medication's efficacy in accelerating rapid fat loss amplifies the visual impact on facial fullness. Although weight-loss-related volume loss can be managed with filler treatments, an incidental observation in a recent study using hyperdilute CaHA-CMC in our office revealed that patients on GLP1RA treated with hyperdilute CaHA-CMC showed unexpected preservation of facial volume.

The purpose of the authors of this retrospective preliminary report is to describe facial-volume outcomes in GLP1RA users treated with hyperdilute CaHA-CMC.

## METHODS

### Study Design

This subanalysis is part of a retrospective cohort study reviewing the medical records of patients treated for mid and lower face rejuvenation between February 2024 and October 2024.^8^ Inclusion criteria included patients presenting with mid and lower face aging changes and fat pad deflation who underwent 2 treatment sessions with hyperdilute CaHA-CMC (1:3 ratio) and completed follow-up visits at 1, 4, and 6 months. Patients were required to provide written informed consent for treatment and inclusion in this retrospective study. Exclusion criteria included incomplete treatment (ie, failure to complete both baseline and 1-month treatment sessions) or incomplete final follow-up (ie, missing 6-month posttreatment evaluation), presence of tattoos or piercings in the treated areas, or receipt of concurrent aesthetic treatments targeting the mid and lower face during the study period. However, this subanalysis particularly analyzed a group of patients in the cohort who were concurrently on GLP1RA that were disclosed only upon completion of the primary study.

### Treatment Protocol

Each syringe of CaHA-CMC contained 1.5 cc of product, with patients receiving 1 syringe per treatment session across 2 sessions spaced 4 weeks apart. To achieve the desired 1:3 dilution, 0.5 cc of 1% lidocaine was added to the CaHA-CMC syringe using a sterile transfer adapter, creating a closed system. The mixture was transferred between the CaHA-CMC syringe and a 10 mL LuerLock syringe for at least 20 passes to ensure homogeneity. The resulting mixture was then connected to another 10 mL syringe containing 4.0 cc of normal saline and passed an additional 20 times to achieve full dilution, yielding 6.0 cc of final product per session.

Before each treatment, topical anesthetic cream (lidocaine/tetracaine 23%/7%) was applied to the treatment areas for 15 min and then removed with alcohol. Injection procedures were performed following the detailed technique described by Durairaj et al.^8^ Briefly, patients were positioned semi-reclined, and the treating physician used a fanning injection technique with a standard 27-G, 0.75-inch needle to deliver the product into targeted anatomical regions, including the subzygomatic fat pad, submalar fat pad, buccal fascial space, and prejowl sulcus. A total of 6.0 cc of diluted CaHA-CMC was injected per session (3.0 cc per side of the face), with ∼1.0 cc delivered into each target zone. Within each zone, ∼5 linear retrograde threads were placed per entry point, with each thread delivering ∼0.05 mL of product, corresponding to roughly 0.25 mL per access site. All treatments were administered by the first author to ensure procedural consistency.

A multilayered technique was employed, primarily delivering the product into the deep dermis at the dermal–fat junction within the subzygomatic fat pad, submalar fat pad, buccal fascial space, and prejowl sulcus. For lower facial areas, intradermal injections were placed along the marionette lines and feathered laterally toward the mandibular and masseteric ligaments using a retrograde linear cross-hatching technique to address fine rhytids and reinforce ligamentous support. Following each treatment session, the treated areas were gently massaged in circular motions to facilitate even product dispersion. Patients were instructed to perform twice-daily self-massage for 5 days post treatment to optimize biostimulatory responses and tissue remodeling.

### Subject Assessment

Standardized and 3-dimensional imaging was conducted using a Nikon D750 camera (Tokyo, Japan) and a QuantifiCare Lifeviz Infinity Pro (QuantifiCare Inc, Suwanee, GA). Volumetric and wrinkle-depth measurements were performed using the QuantifiCare LifeViz Infinity Pro system (QuantifiCare Inc), which provides calibrated 3-dimensional reconstructions from stereophotogrammetric images. Cheek and jowl volumes were calculated using the system's anatomical region segmentation algorithm, with changes expressed in milliliters. Nasolabial fold (NLF) and marionette-line depths were assessed using the system's automated wrinkle analysis module, which quantifies average linear depression (in millimeters) across standardized tracing zones from baseline to follow-up. All analyses were performed on matched image sets under identical lighting and positioning conditions. The treating physician and a secondary evaluator (an aesthetic nurse practitioner blinded to the patients’ GLP1RA status) assessed each patient at each time point (baseline, 1, 4, and 6 months) according to the 5-point Global Aesthetic Improvement Scale (GAIS). Similarly, patients self-evaluated according to GAIS at each time point and recorded their satisfaction with the treatment according to a 5-point patient satisfaction survey (1 = extremely dissatisfied, 3 = neutral, 5 = extremely satisfied). A comprehensive physical examination was conducted at every follow-up to document wellness, monitor for side effects, and address any patient concerns. Body weight was recorded at each time point based on patient self-reporting.

### Statistical Analysis

Weight loss was recorded in pounds and expressed as the average ± standard deviation percent change from baseline to Month 6. A simple linear regression tested for significant nonzero slopes.

Ordinal data were summarized as the percentage of patients in each category. Empirical measurements (eg, cheek volume, jowl volume, NLF depth, and marionette wrinkle depth) were analyzed using paired 1-way analysis of variance followed by Dunnett's multiple comparisons test. Individual patient data were graphed, and total volume and average fold/wrinkle depths were calculated by averaging measurements from the left and right sides of the face at each time point.

One patient did not undergo scanning at Month 4; their values were imputed using quadratic curve fits for comparisons requiring averaged data. Correlation analyses between the percentage changes of each measured variable were conducted using Pearson's correlation coefficient (*r*) and associated *P*-values. Statistical analyses were performed using GraphPad Prism version 10.4.0 (GraphPad Software, Boston, MA). Significance was defined as *P* < .033, and ns represented *P* > .033.

## RESULTS

### Patient Characteristics

**Figure 1.**
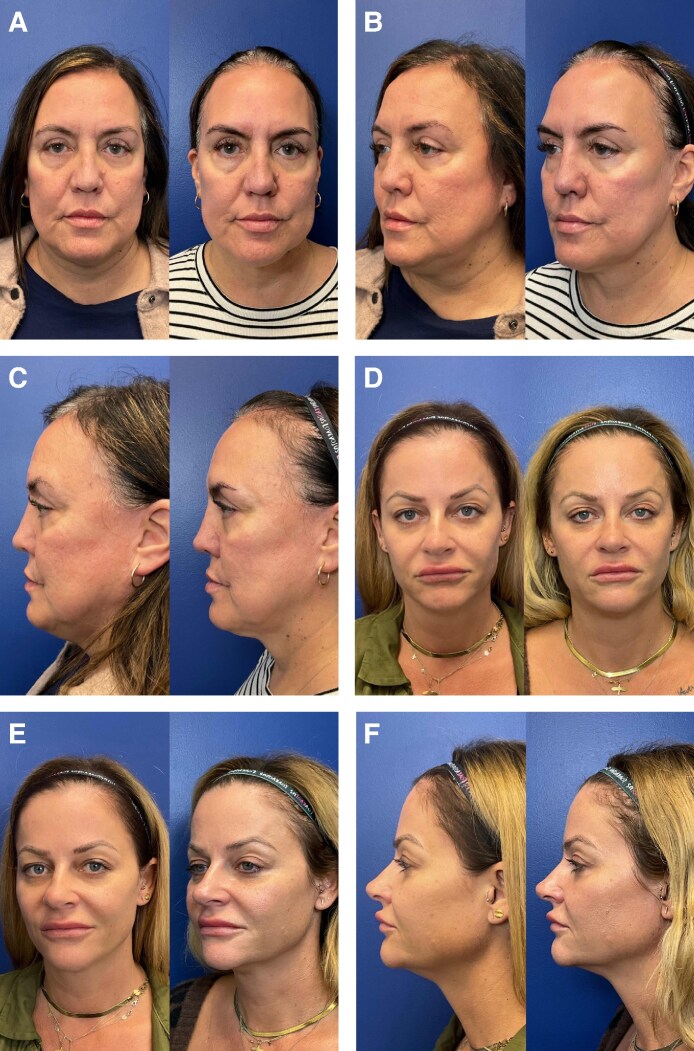
Before and 6 months after hyperdilute CaHA-CMC during concurrent weight loss. (A) Before and (B) 6 months after, front view; (C) before and (D) 6 months after, orthogonal view; and (E) before and (F) 6 months after, profile view of a 54-year-old patient who lost approximately 36 pounds during the study. (G) Before and (H) 6 months after, front view; (I) before and (J) 6 months after, orthogonal view; and (K) before and (L) 6 months after, profile view of a 45-year-old patient who lost approximately 34 pounds during the study.

This subanalysis included 4 female patients aged 45 to 54 (mean age = 50.25 ± 4.1 years). All patients had been on GLP1RA for 6 to 12 months and reported significant weight loss. None of the 4 patients included in this analysis had a documented history of Type 2 diabetes mellitus or insulin resistance, and all were using GLP1RAs for weight loss. The 4 patients were otherwise generally healthy with normal lifestyles and diets. GLP1RAs included Zepbound (tirzepatide, Eli Lilly and Company, Indianapolis, IN), Mounjaro (tirzepatide, Eli Lilly and Company), Saxenda (liraglutide, Novo Nordisk), and Ozempic. At baseline, patients were receiving the following GLP1RA doses: Zepbound 2.5 mg/week, Mounjaro 5 mg/week, Saxenda 3 mg/day, and Ozempic 0.2 mg/week. The average time on GLP1RA therapy before baseline was ∼2.2 months, ranging from 12 days to 3.5 months. By 6 months, 2 patients had undergone dose escalation (Zepbound 5 mg/week, Ozempic 1 mg/week), whereas 2 patients (Mounjaro, Saxenda) remained at their original doses. Patient characteristics and GLP1RA dosing are reported in Table 1. Despite substantial global weight loss ranging from 11 to 36 pounds, none reported visible facial-volume depletion following treatment with hyperdilute CaHA-CMC (Figure 1, Supplemental Figures 1-19).

**Table 1.** Patient Characteristics and GLP1RA Dosing Regimen Throughout the Study

					Baseline			Month 1			Month 4			Month 6		
Patient ID	Age (years)	Height (in)	GLP1RA brand	Approximate time on GLP1RA before baseline	Weight (lb)	BMI	Dose (mg)	Weight (lb)	BMI	Dose (mg)	Weight (lb)	BMI	Dose (mg)	Weight (lb)	BMI	Dose (mg)
MM	54	67	Zepbound	12 days	263	41.2	2.5	242	37.9	2.5	240	37.6	2.5	227	35.5	5.0
GL	49	67	Mounjaro	1.5 months	171	26.8	5.0	168	26.3	5.0	160	25.1	5.0	160	25.1	5.0
VW	53	67	Saxenda	3.5 months	234	36.6	3.0	225	35.2	3.0	220	34.5	3.0	218	34.1	3.0
HT	45	66	Ozempic	3.5 months	162	26.1	0.2	128	20.7	0.2	128	20.7	1.0	128	20.7	1.0

GLP1RA, glucagon-like peptide-1 receptor agonist.

### Weight Loss

**Figure 2.**
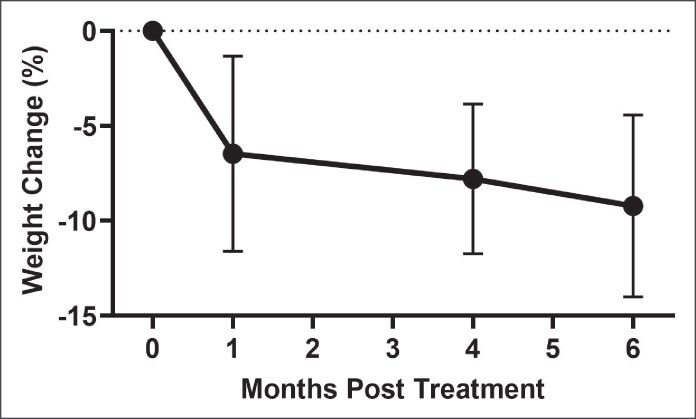
Average percent change in body weight of patients on GLP1RAs.

Patients undergoing GLP1RA treatment reported losing an average of 24.25 lb throughout their time enrolled in this study (maximum weight lost: 36 lb; minimum weight lost: 11 lb) (*P* = .0651). This trend in weight loss appeared to stabilize by 4 months post treatment. The average percentage body weight lost 1, 4, and 6 months post treatment (compared with baseline) was 6.46% ± 5.15%, 7.80% ± 3.93%, and 9.22% ± 4.79%, respectively (Figure 2, Supplemental Figure 20). Although this did not reach statistical significance because of the small sample size, such dramatic and short-term weight loss was considered clinically significant by the providers. Mean BMI significantly decreased progressively across visits: 32.7 ± 6.6 kg/m^2^ at baseline, 30.0 ± 6.6 kg/m^2^ at 1 month, 29.5 ± 6.3 kg/m^2^ at 4 months, and 28.9 ± 6.2 kg/m^2^ at 6 months (*P* = .032). All patients exhibited reductions in weight and BMI during the study period.

### Facial Volumization

**Figure 3.**
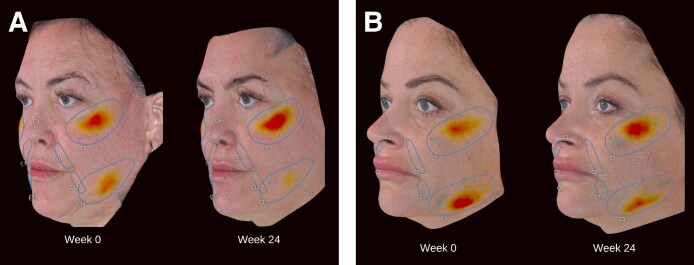
Three-dimensional volumetric scans (A) before and (B) 6 months after hyperdilute CaHA-CMC treatment during concurrent weight loss in a 54-year-old patient who lost approximately 36 pounds. Three-dimensional volumetric scans (C) before and (D) 6 months after hyperdilute CaHA-CMC treatment during concurrent weight loss in a 45-year-old patient who lost approximately 34 pounds. Heat maps indicate soft-tissue displacement, with red and orange representing areas of greatest outward projection and pale blue denoting minimal or no change.

**Figure 4.**
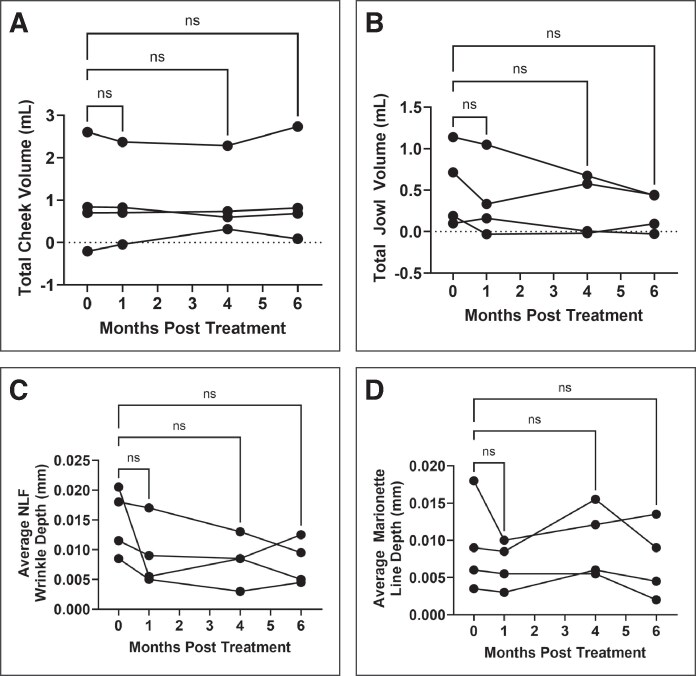
Total (A) cheek and (B) jowl volume measured at baseline and 1-, 4-, and 6-months post-treatment. Average (C) nasolabial fold and (D) marionette wrinkle depth measured at baseline and 1-, 4-, and 6-months post-treatment.

Facial-volume scans were used to objectively assess the volume of the cheeks and jowls over time (Figure 3A, B). No significant changes in total cheek volume, left cheek volume, or right cheek volume were detected at any time point (*P* = .9105, *P* = .6895, and *P* = .8132, respectively; Figure 4A, Supplemental Figure 21A, B). Mean total cheek volume increased 9.77% from pretreatment (0.985 mL) to 6 months posttreatment (1.081 mL). Overall, cheek volume was stable with no significant linear trend over time (*P* = .9712).

Similarly, no significant changes in total jowl, left jowl, or right jowl volume were detected at any time point (*P* = .6528, *P* = .6895, *P* = .8106, respectively; Figure 4B, Supplemental Figure 22A, B). Mean total jowl volume decreased 55.83% from pretreatment (0.5350 mL) to 6 months post treatment (0.2363 mL). Overall, jowl volume steadily decreased over time, although this trend was not statistically significant (*P* = .2614).

### Facial Wrinkle Depths

In addition to facial-volume measures, NLF and marionette wrinkle depths were measured at each time point. No significant changes in average NLF, left NLF, or right NLF depth were detected at any time point (*P* = .1655, *P* = .4062, *P* = .1605, respectively; Figure 4C, Supplemental Figure 23A, B). Average NLF depth improved, decreasing 46.17% from 0.015 mm pretreatment to 0.008 mm 6 months post treatment. Average NLF depth steadily decreased over time, although this trend did not reach statistical significance (*P* = .0866).

Additionally, no significant changes in average marionette, left marionette, or right marionette wrinkle depths were detected at any time point (*P* = .8880, *P* = .3350, *P* = .9084, respectively) (Figure 4D, Supplemental Figure 24A, B). Average marionette wrinkle depth decreased 20.55% from pretreatment (0.009 mm) to 6 months post treatment (0.007 mm). Although marionette depth generally improved over time, 1 patient exhibited a transient increase at 4 months before returning to baseline trends, although this trend was also not statistically significant (*P* = .0866).

### Facial Vector Analysis

QuantifiCare vector analysis demonstrated consistent superior and lateral soft-tissue displacement across all patients from baseline to 6 months post treatment, indicating preserved midface volume and lower face contouring despite concurrent weight loss. The most pronounced vector displacement was observed in the patients that lost the least (11 pounds) and most (36 pounds) weight, with both exhibiting strong superior and lateral vectors in the midface, suggesting effective volumization and structural support (Supplemental Figure 25A, B). Jowl region vectors indicated upward displacement in all patients. Across all cases, vector magnitude correlated with visible soft-tissue support, with superior displacement vectors in the cheek and lower face, suggesting reductions in skin laxity and maintained midface projection.

### Relationships Between Weight Loss and Facial Aesthetic Changes

**Figure 5.**
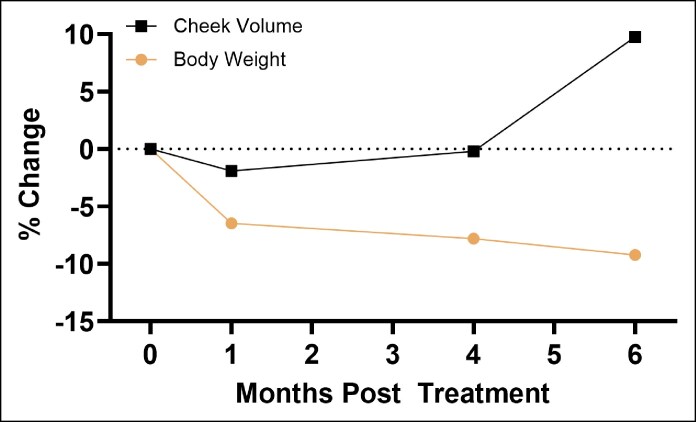
Mean percent change in cheek volume and body weight over the course of the study.

Despite GLP1RA-induced weight loss, there were no significant correlations between cheek volume (*P* = .518) or marionette wrinkle depth (*P* = .637) changes and weight loss (Figure 5). However, significant correlations existed between weight loss and NLF depth (*P* = .008) and jowl volume (*P* = .017) changes. Directionally, cheek volume was inversely correlated with weight loss, whereas jowl volume, NLF depth, and marionette wrinkle depth were positively correlated with weight loss (Supplemental Figure 26). Jowl volume, NLF wrinkle depth, and marionette depth were all negatively correlated with cheek volume, although these correlations did not reach statistical significance. The overall percentage changes in each measure are presented in Table 2.

**Table 2.** Percent Changes in Body Weight, Cheek Volume, Jowl Volume, Nasolabial-Fold Depth, and Marionette Wrinkle Depth

Time post treatment	Body weight (% change)	Cheek volume (% change)	Jowl volume (% change)	Nasolabial fold depth (% change)	Marionette wrinkle depth (% change)
1 Month	−6.46	−1.91	−29.57	−37.63	−26.03
4 Months	−7.79	−0.20	−42.26	−43.61	7.12
6 Months	−9.22	9.77	−55.83	−46.17	−20.55

### Patient Satisfaction and Global Aesthetic Scale Evaluation

Patient satisfaction showed a notable increase over time, demonstrating progressive improvement in perceived outcomes at 1, 4, and 6 months post treatment (Supplemental Figure 27). At 1 month, 50% of patients reported being at least satisfied, with 25% indicating satisfaction and 25% very high satisfaction. By 4 months, 50% of patients were very satisfied, whereas the other half remained neutral. At 6 months, satisfaction peaked, with 100% of patients being at least satisfied, including 75% who were very satisfied and 25% satisfied. Importantly, no patients reported dissatisfaction at any time point.

**Figure 6.**
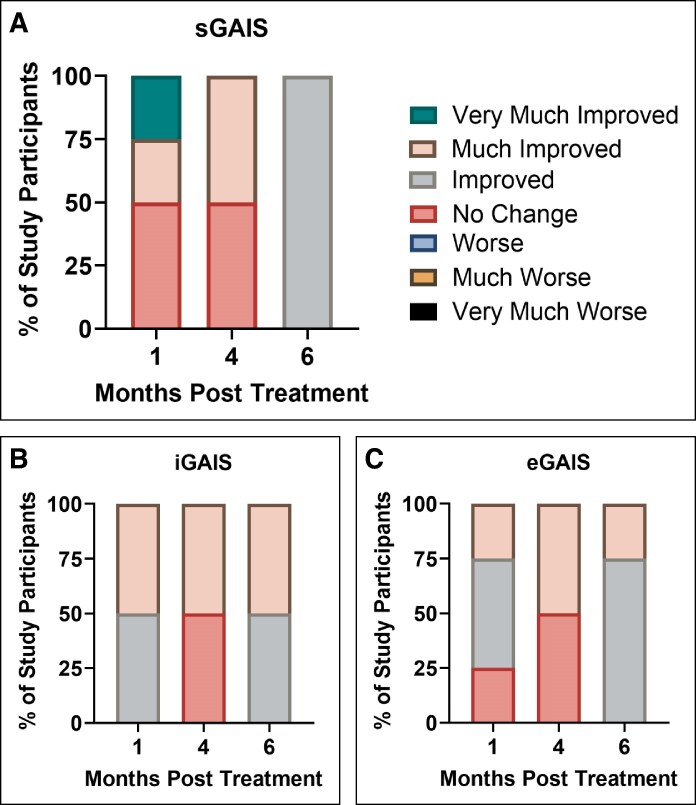
Global Aesthetic Improvement Scores according to (A) subjects (sGAIS), (B) the principal investigator (iGAIS), and (C) a blinded evaluator (eGAIS).

Patient-reported GAIS (sGAIS) closely mirrored patient satisfaction results and reflected a growing sense of self-perceived improvement over time (Figure 6A). At 1 month, 50% of patients were at least improved, with 25% much improved and 25% very much improved, whereas the other half reported no change. By 4 months, 50% of patients were much improved, whereas the remaining 50% saw no change. By 6 months, all patients (100%) reported being at least improved, with no reports of worse outcomes or no change, marking a complete and progressive improvement in self-reported satisfaction.

Investigator-assessed outcomes (iGAIS) mirrored these positive trends (Figure 6B). At 1 month, all patients (100%) were rated as at least improved, with 50% classified as improved and 50% as much improved. At 4 months, ratings shifted slightly, with 50% of patients showing no change and the other 50% rated as much improved. By 6 months, all patients were rated as at least improved, evenly split between improved (50%) and much improved (50%). No patients were rated as worse throughout the study period.

Blinded evaluator assessments (eGAIS) also indicated sustained improvement (Figure 6C). At 1 month, 75% of patients were rated as at least improved, with 50% improved and 25% much improved, whereas 25% showed no change. By 4 months, half of the patients remained unchanged, whereas 50% were rated as much improved. By 6 months, 100% of patients were rated as at least improved, with 75% improved and 25% much improved. No patients were rated as worse throughout the study period.

### Safety

No adverse events or complications were observed in the included patients throughout the treatment or follow-up period.

## DISCUSSION

This case series provides preliminary evidence that hyperdilute CaHA-CMC may mitigate facial-volume loss in patients experiencing rapid weight loss because of GLP1RA agonist use. Rapid weight loss commonly results in significant reductions in subcutaneous fat and supportive dermal structures, contributing to the phenomenon colloquially known as Ozempic Face. Despite their substantial average weight loss, patients in this study exhibited preserved facial volume and stable facial contours as objectively measured and corroborated by both patient and investigator assessments.

The volumetric analysis revealed no statistically significant changes in cheek or jowl volume over 6 months. However, trends indicating slight increases in cheek volume and reductions in jowl volume may reflect localized improvements in facial structure, attributable to the biostimulatory effects of CaHA-CMC and corroborating findings by Rovatti et al.^3^ For example, it is conceivable that reductions in jowl volume are partially attributable to both weight loss and subtle cheek volumization. These findings align with previous evidence demonstrating that hyperdilute CaHA-CMC stimulates ECM production, particularly collagen and elastin, which may counteract weight-loss-related soft-tissue atrophy, enhance dermal support, and tighten skin.^2,9,10^ Furthermore, improvements in NLF and marionette wrinkle depths were observed despite weight loss, although these did not reach statistical significance, likely because of the small sample size. The observed improvements highlight the potential of CaHA-CMC to retain facial volume, facilitate skin tightening, and combat wrinkles that may have otherwise worsened during weight loss.^11^ These observations are further supported by vector analysis, which demonstrated superior and lateral soft-tissue displacement in all patients, consistent with preserved midface volume and lower face contouring. Jowl region vectors also showed an upward displacement across all cases, indicating improvements in skin laxity and structural support. These results align with volumetric trends observed in the study, reinforcing the hypothesis that hyperdilute CaHA-CMC supports facial structure and mitigates weight-loss-associated volume depletion.

Patient satisfaction and self-assessed GAIS ratings demonstrated a progressive increase over time, with all patients reporting high levels of satisfaction and aesthetic improvement by 6 months. This time-dependent effect aligns with the gradual collagen-building process commonly noted with biostimulator use.^12,13^ Blinded evaluator and investigator ratings further support these findings and support the use of hyperdilute CaHA-CMC for facial-volume preservation. This is particularly noteworthy given the challenges of achieving high satisfaction in patients concurrently undergoing rapid and visible physical transformations without a surgical intervention.

Interestingly, although no significant correlations were detected between weight loss and changes in cheek volumes, significant positive correlations were observed between weight loss and reductions in NLF depth and jowl volume. In addition, jowl volume, NLF depth, and marionette wrinkle depth were all inversely correlated with cheek volume. These findings suggest that weight loss and cheek volumization may both partially contribute to reducing jowl volume and facial wrinkles. Further research is needed to explore the regional effect of ECM-stimulating treatments and weight loss on these facial aesthetic changes.

From a clinical-practice perspective, these observations favor an early or proactive intervention model rather than a wait-and-correct approach. In our practice, we recommend initiating biostimulatory treatment with hyperdilute CaHA-CMC after patients have lost ∼5% of their baseline body weight, but before clinically visible midfacial hollowing occurs. This timing allows for early ECM stimulation, with the goal of offsetting subcutaneous fat loss before overt aesthetic changes become pronounced. Anecdotally, patients managed using this preventive algorithm have not subsequently required structural volumizing fillers, presumably because early neocollagenesis provides a supportive scaffold that helps preserve facial contours as weight loss progresses.

Prospective, controlled trials are warranted to confirm the optimal timing, efficacy, and cost-effectiveness of this preventive strategy, but the present data offer an initial framework for integrating early collagen biostimulation into the aesthetic management of GLP1RA users. The implications of these findings are significant for aesthetic medicine, particularly in addressing the unique challenges posed by weight-loss-induced facial changes in GLP1RA users.^14^ By preserving facial volume and improving dermal support, hyperdilute CaHA-CMC offers a promising option for mitigating the aesthetic consequences of rapid weight loss.

### Limitations

This study's limitations, including its small sample size, retrospective design, self-reported patient weights, limited generalizability due to variation in GLP1RA types and dosing regimens, and lack of a control group, warrant cautious interpretation of these results. In addition, 1 patient demonstrated an anomalous (although not significant) increase in marionette-line depth at Month 4, likely reflecting limitations of stereophotogrammetry-based analysis, including sensitivity to minor changes in facial expression, lighting, or head positioning rather than true anatomical change. Larger, prospective studies with randomized controls are necessary to validate these observations and optimize treatment protocols, including dilution ratios, injection techniques, and timing relative to weight loss trajectories.

## CONCLUSIONS

This case series suggests that hyperdilute CaHA-CMC may preserve facial volume and improve aesthetics in patients experiencing rapid weight loss with GLP1RA. Despite significant weight loss, patients demonstrated stable facial volumes and high satisfaction rates, likely because of the biostimulatory effects of CaHA-CMC. These findings warrant further studies to validate the results and optimize treatment protocols. Hyperdilute CaHA-CMC offers a promising approach to addressing weight-loss-related facial changes with collagen stimulation, enhancing outcomes in regenerative aesthetics.

## Supplemental Material

This article contains supplemental material located online at https://doi.org/10.1093/asjof/ojaf088.

## Supplementary Material

ojaf088_Supplementary_Data
