# Supplementary material for: Hyperdilute Radiesse Preserves Facial Volume in Glucagon-Like Peptide-1 Receptor Agonist Users Undergoing Rapid Weight Loss
Source: Aesthet Surg J Open Forum. 2025 Oct 21;7:ojaf088. doi: 10.1093/asjof/ojaf088 (PMC12538281; doi:10.1093/asjof/ojaf088)
Supplement: ojaf088_Supplementary_Data [file ojaf088_supplementary_data.docx]

**Supporting Information**

**Hyperdilute Radiesse® for Facial-Volume Preservation in GLP-1 Receptor Agonist Users Undergoing Rapid Weight Loss: A Preliminary Report**

**
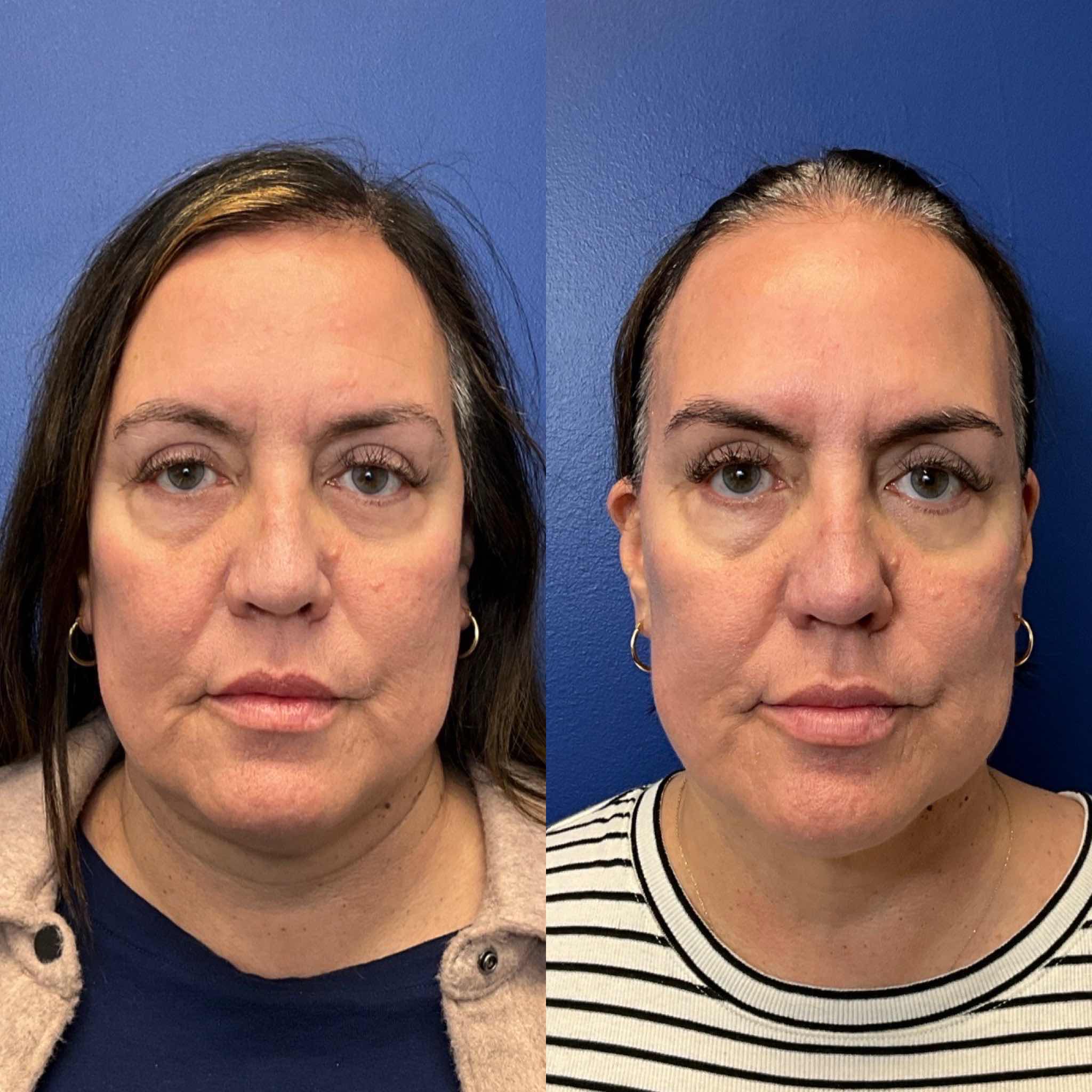
**

**Figure S1. Before and 6 months after hyperdilute CaHA-CMC during concurrent weight loss in a 54-year-old patient who lost approximately 36 pounds during the study.**

**
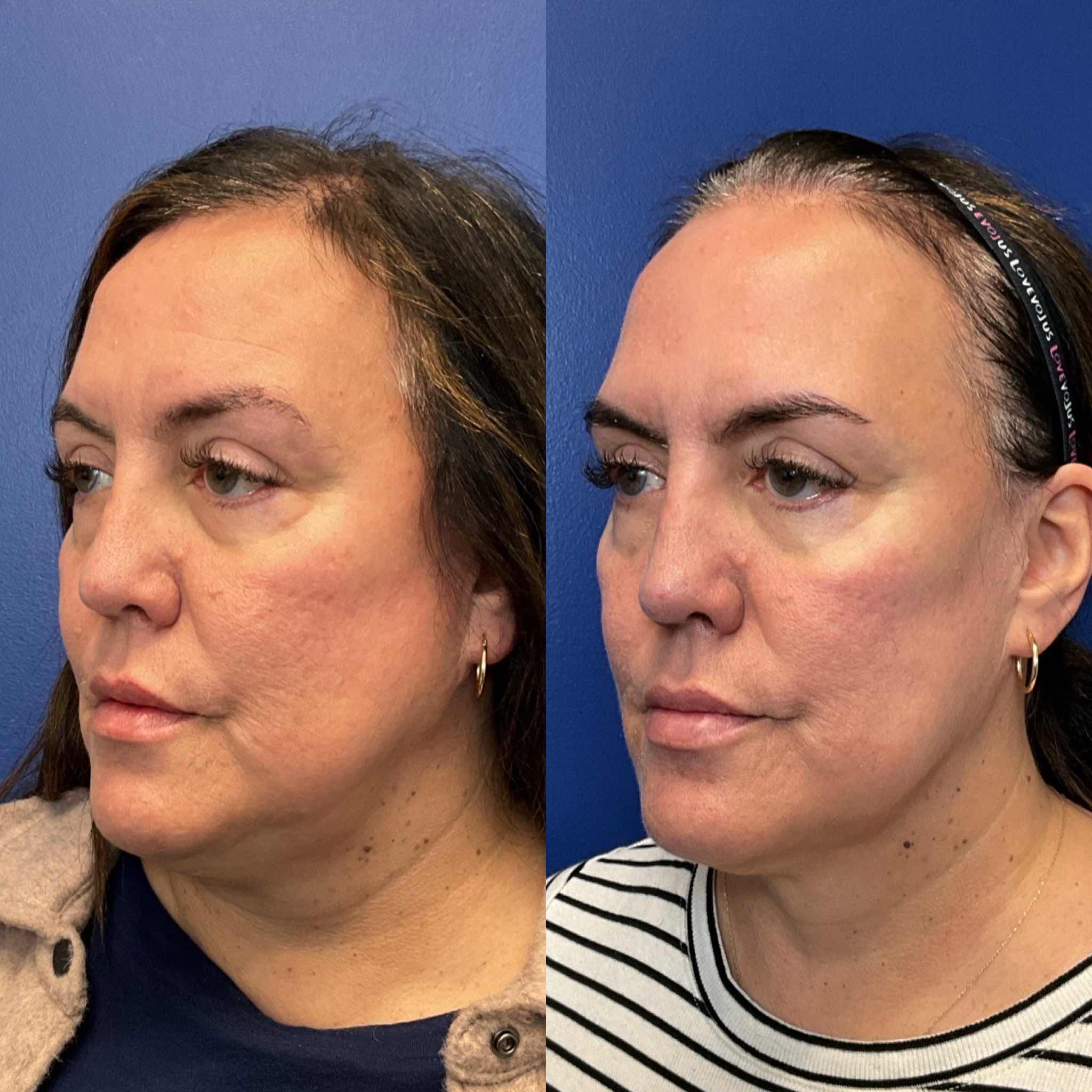
**

**Figure S2. Before and 6 months after hyperdilute CaHA-CMC during concurrent weight loss in a 54-year-old patient who lost approximately 36 pounds during the study.**

**
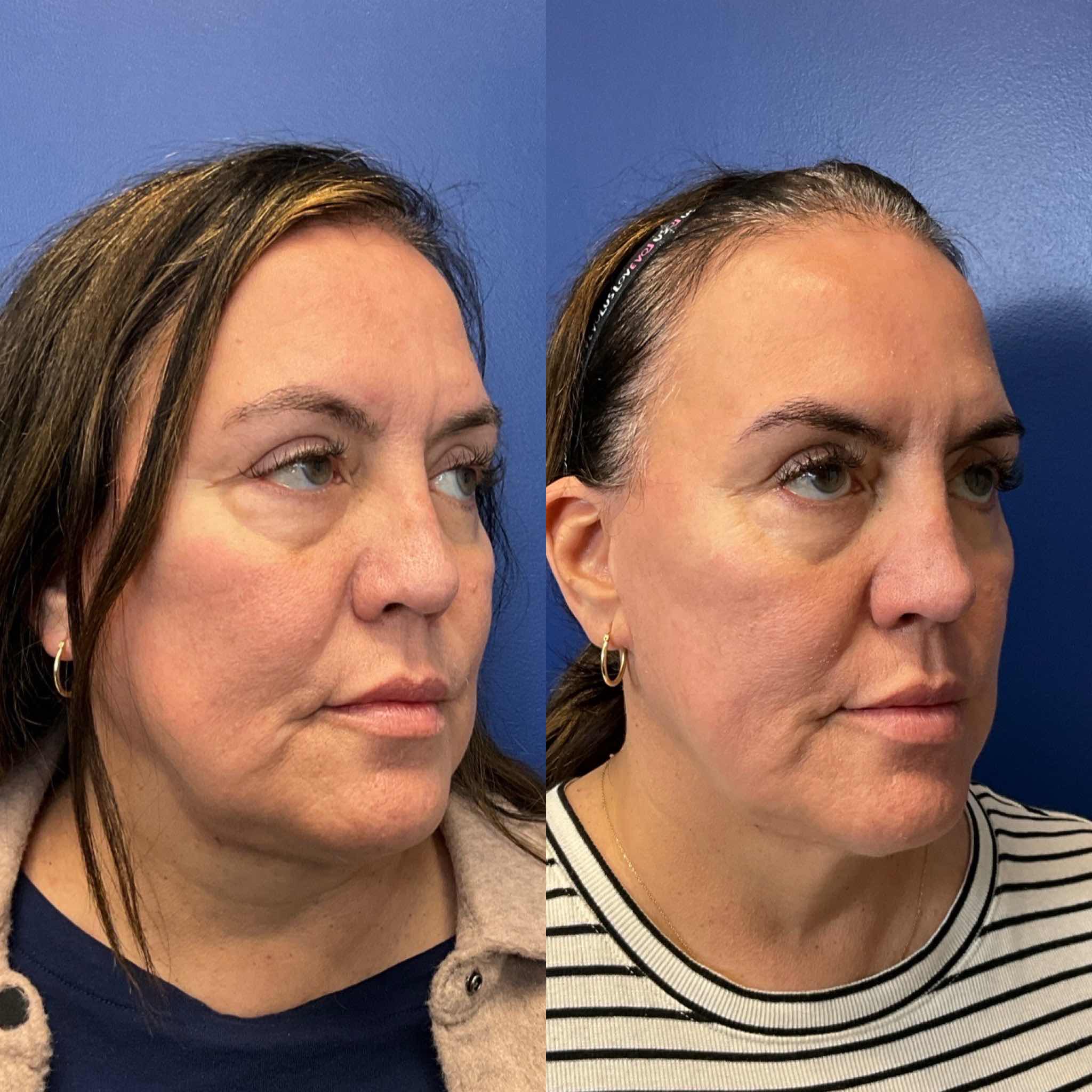
**

**Figure S3. Before and 6 months after hyperdilute CaHA-CMC during concurrent weight loss in a 54-year-old patient who lost approximately 36 pounds during the study.**

**
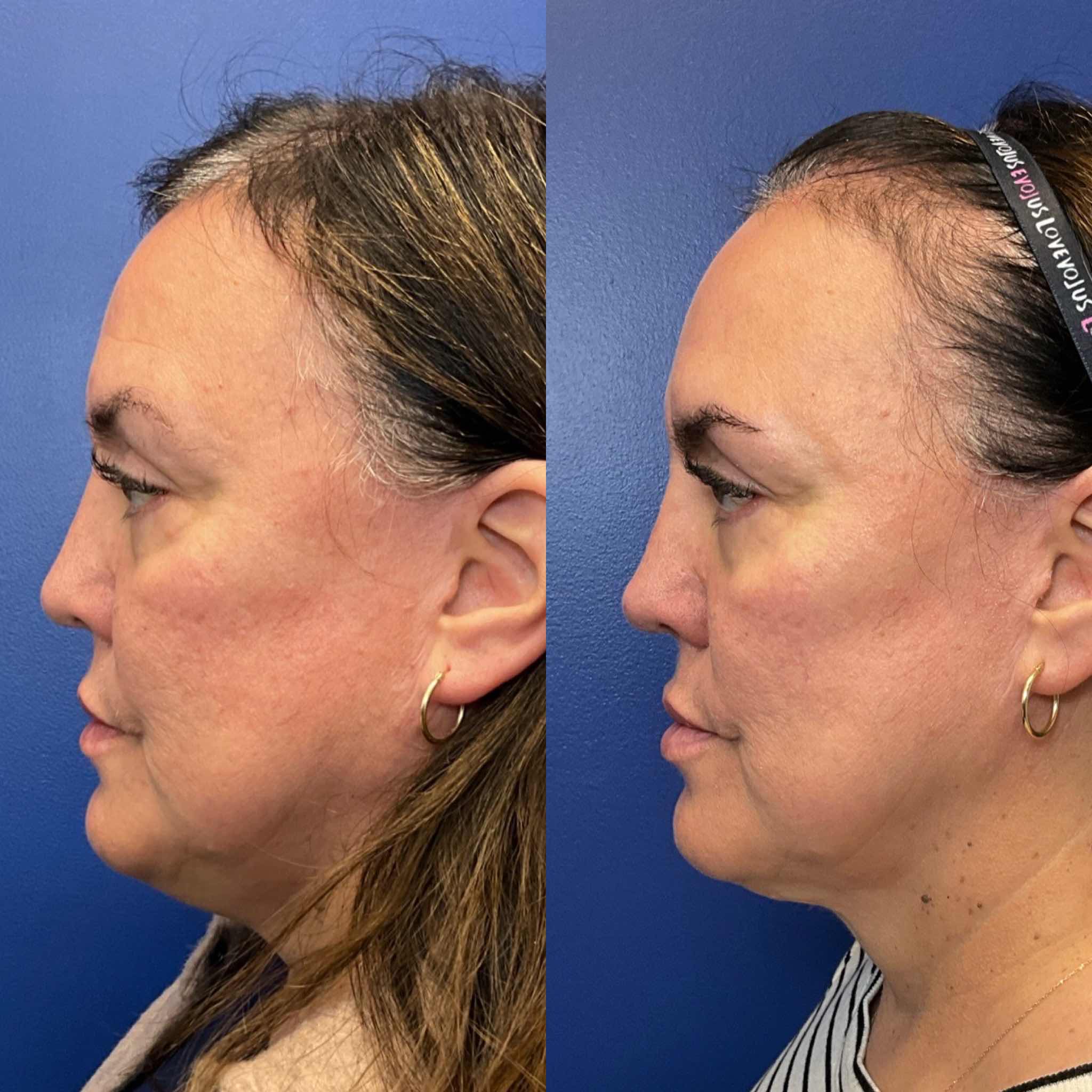
**

**Figure S4. Before and 6 months after hyperdilute CaHA-CMC during concurrent weight loss in a 54-year-old patient who lost approximately 36 pounds during the study.**

**
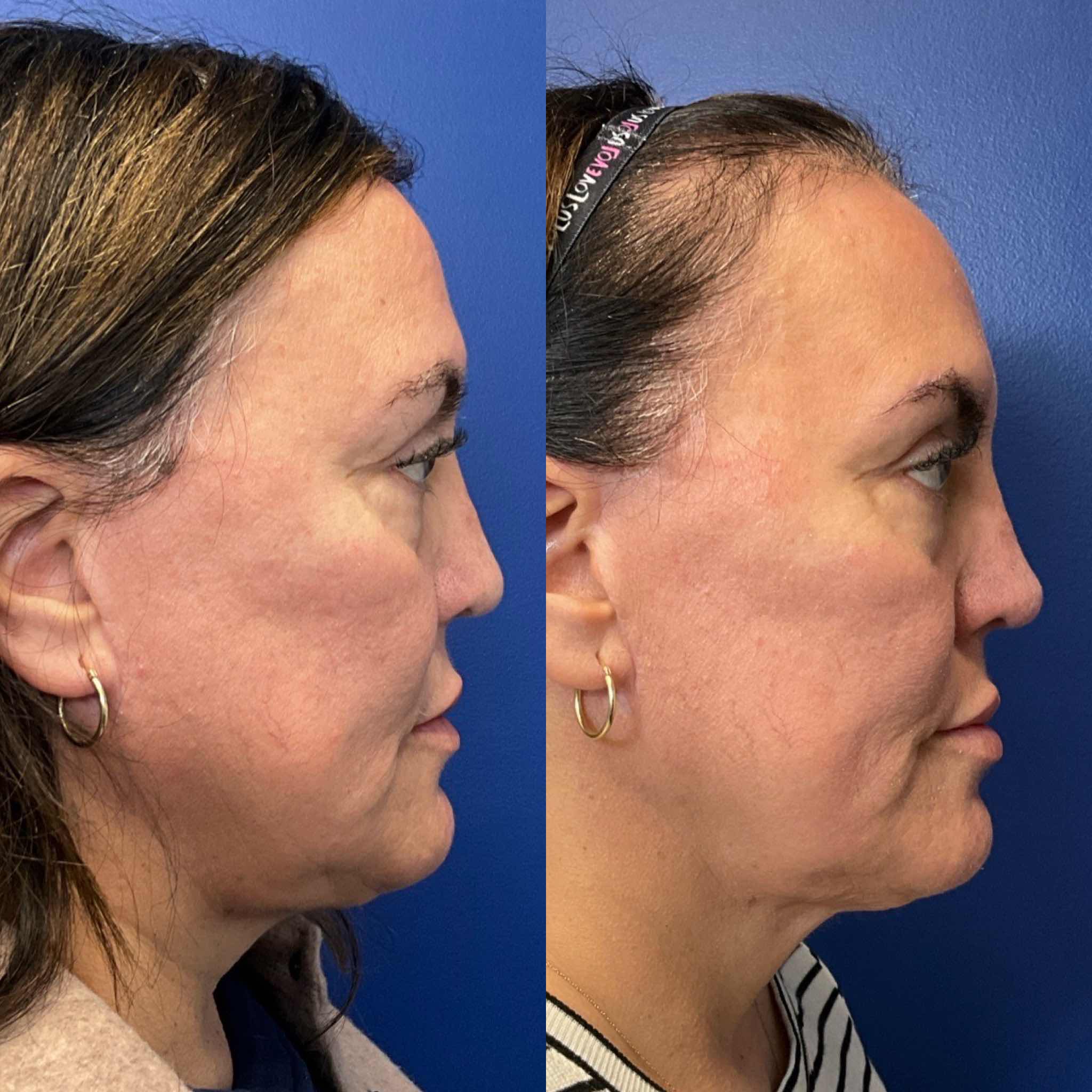
**

**Figure S5. Before and 6 months after hyperdilute CaHA-CMC during concurrent weight loss in a 54-year-old patient who lost approximately 36 pounds during the study.**

**
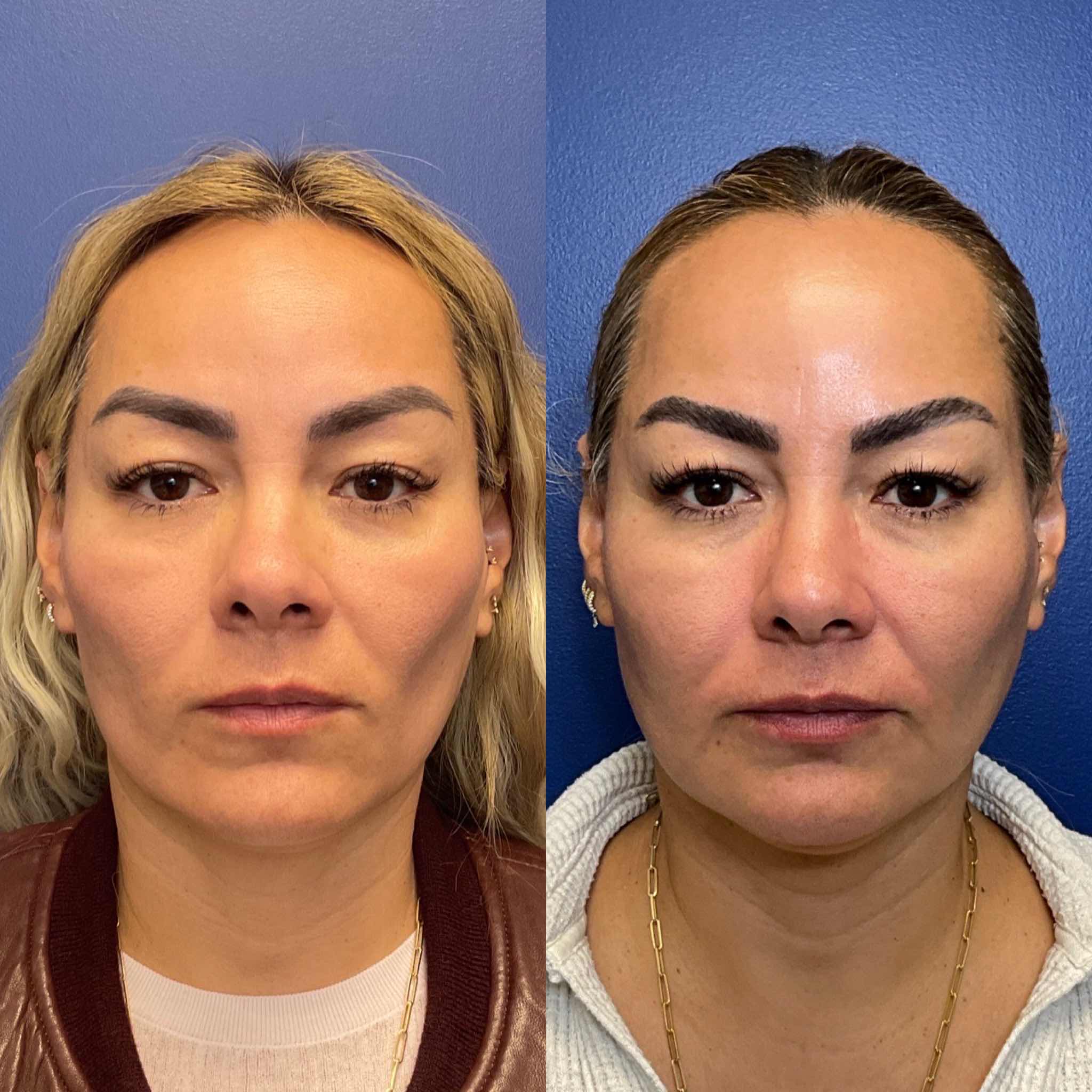
**

**Figure S6.** **Before and 6 months after hyperdilute CaHA-CMC during concurrent weight loss in a 49-year-old patient who lost approximately 11 pounds during the study.**

**
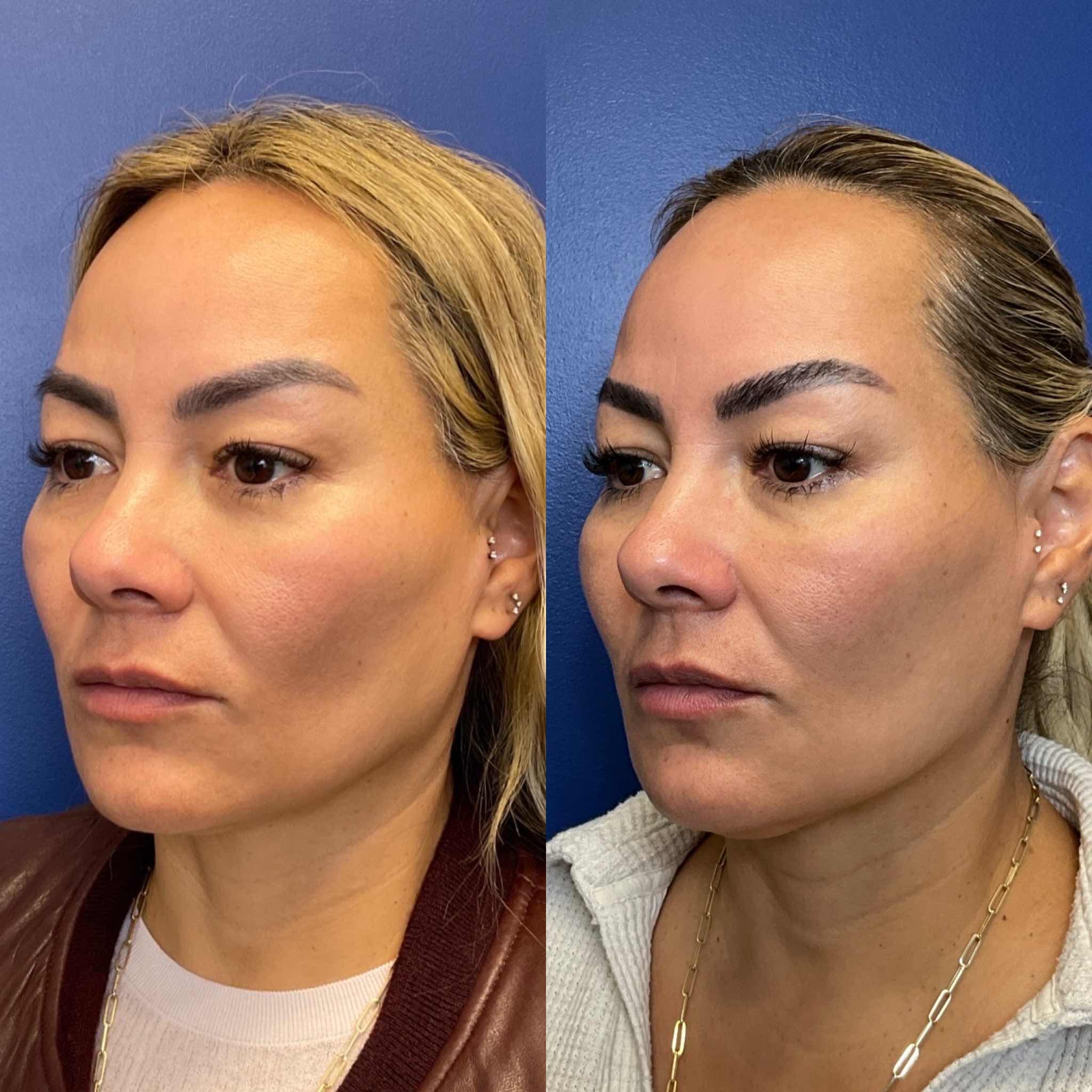
**

**Figure S7. Before and 6 months after hyperdilute CaHA-CMC during concurrent weight loss in a 49-year-old patient who lost approximately 11 pounds during the study.**

**
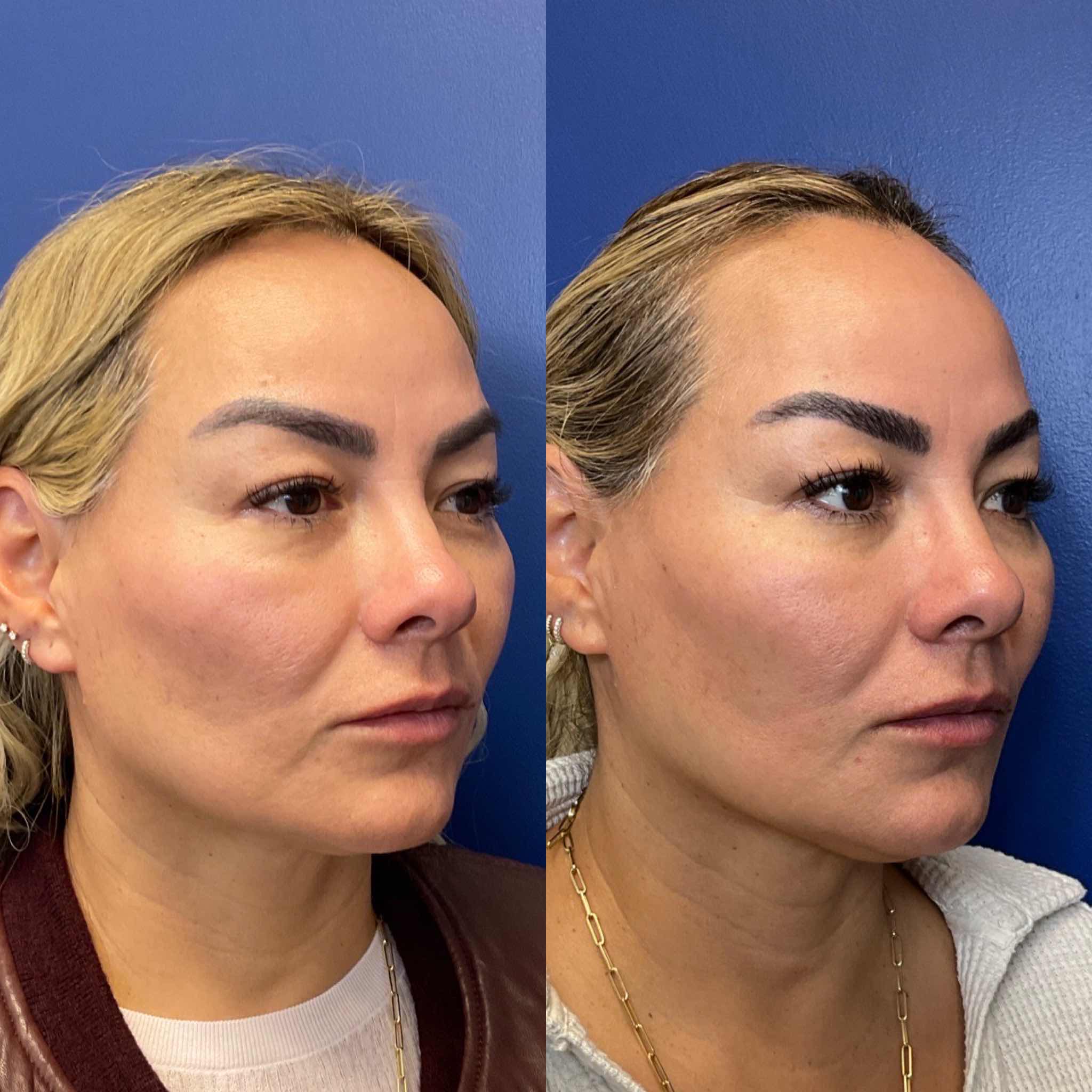
**

**Figure S8. Before and 6 months after hyperdilute CaHA-CMC during concurrent weight loss in a 49-year-old patient who lost approximately 11 pounds during the study.**

**
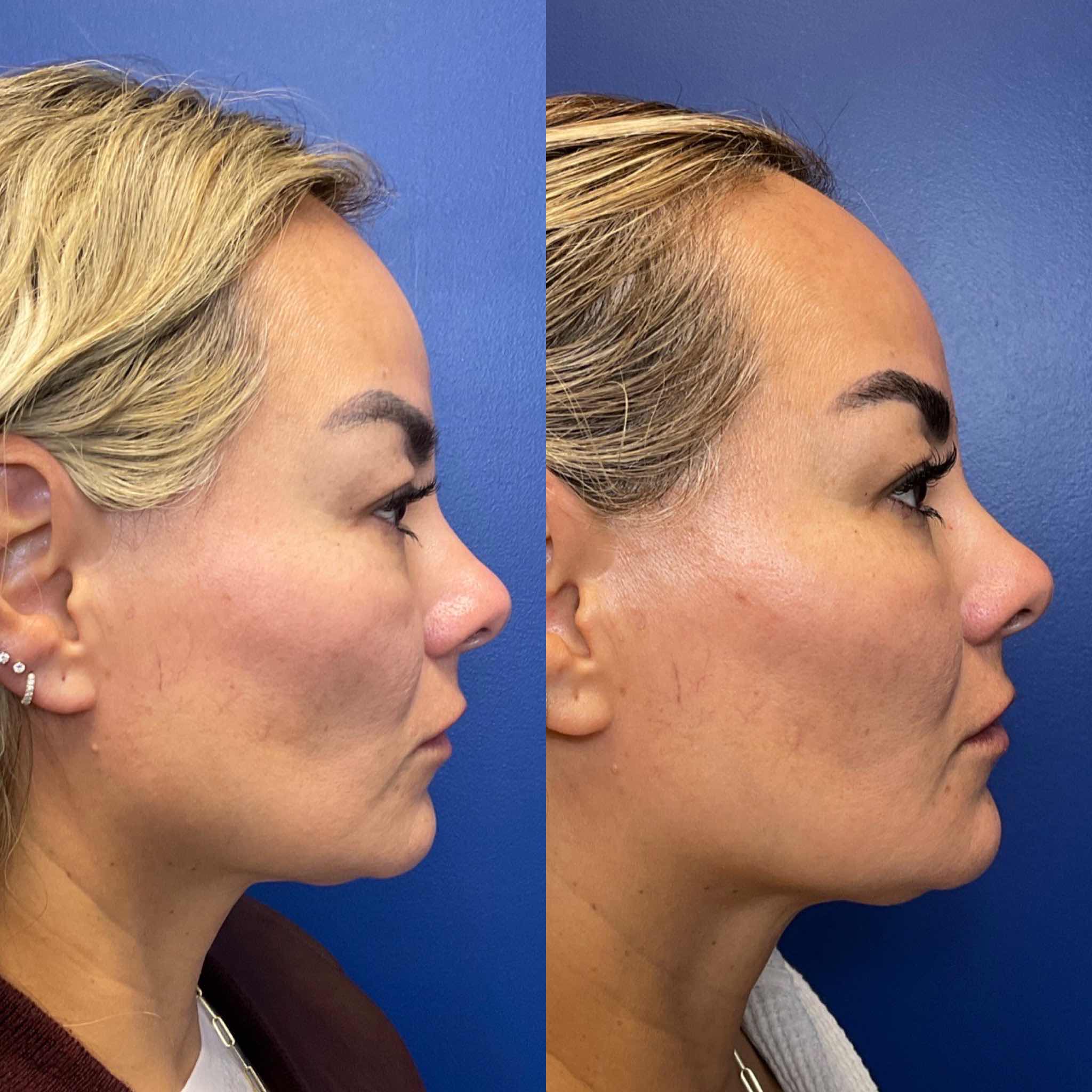
**

**Figure S9. Before and 6 months after hyperdilute CaHA-CMC during concurrent weight loss in a 49-year-old patient who lost approximately 11 pounds during the study.**

**
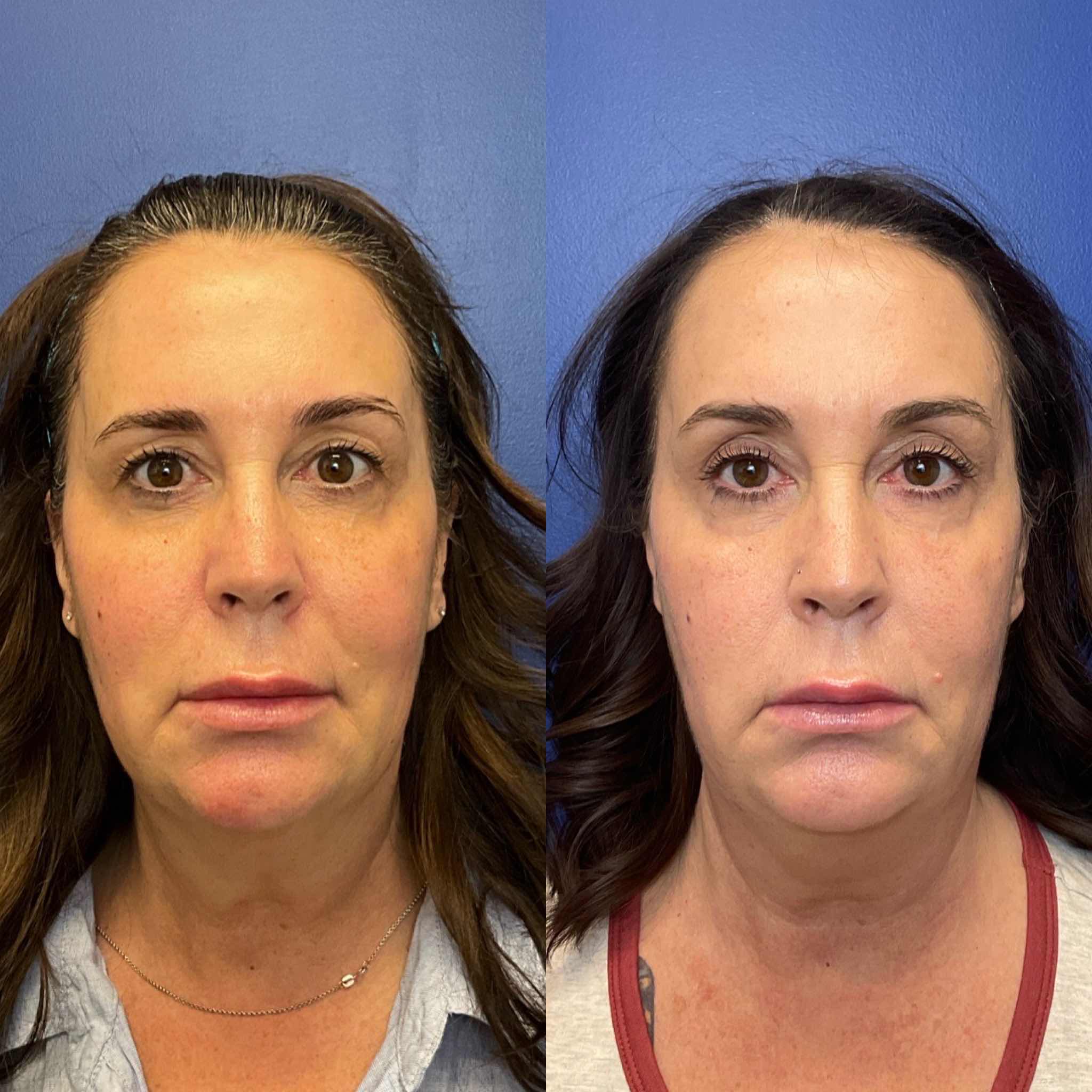
**

**Figure S10. Before and 6 months after hyperdilute CaHA-CMC during concurrent weight loss in a 53-year-old patient who lost approximately 16 pounds during the study.**

**
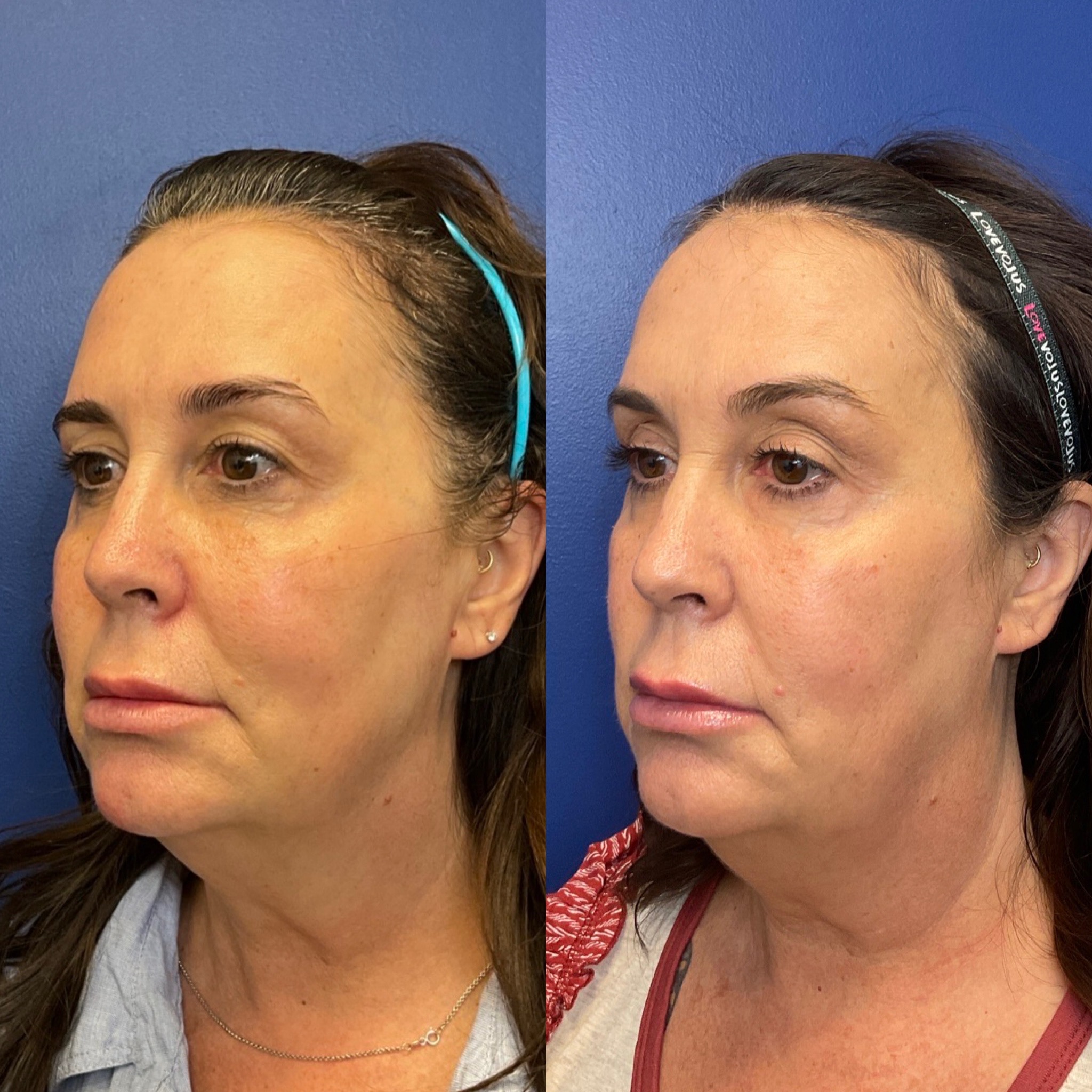
**

**Figure S11. Before and 6 months after hyperdilute CaHA-CMC during concurrent weight loss in a 53-year-old patient who lost approximately 16 pounds during the study.**

**
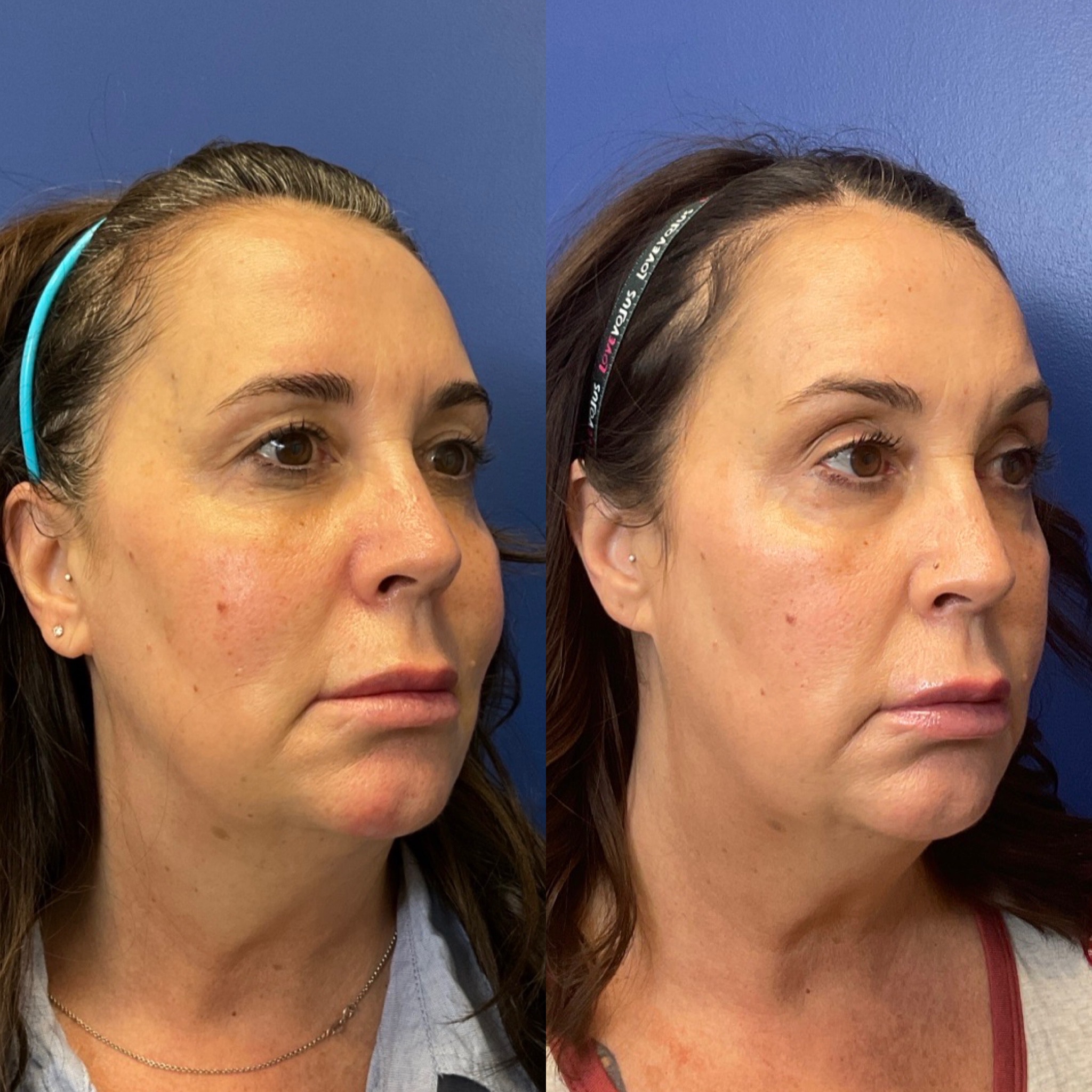
**

**Figure S12. Before and 6 months after hyperdilute CaHA-CMC during concurrent weight loss in a 53-year-old patient who lost approximately 16 pounds during the study.**

**
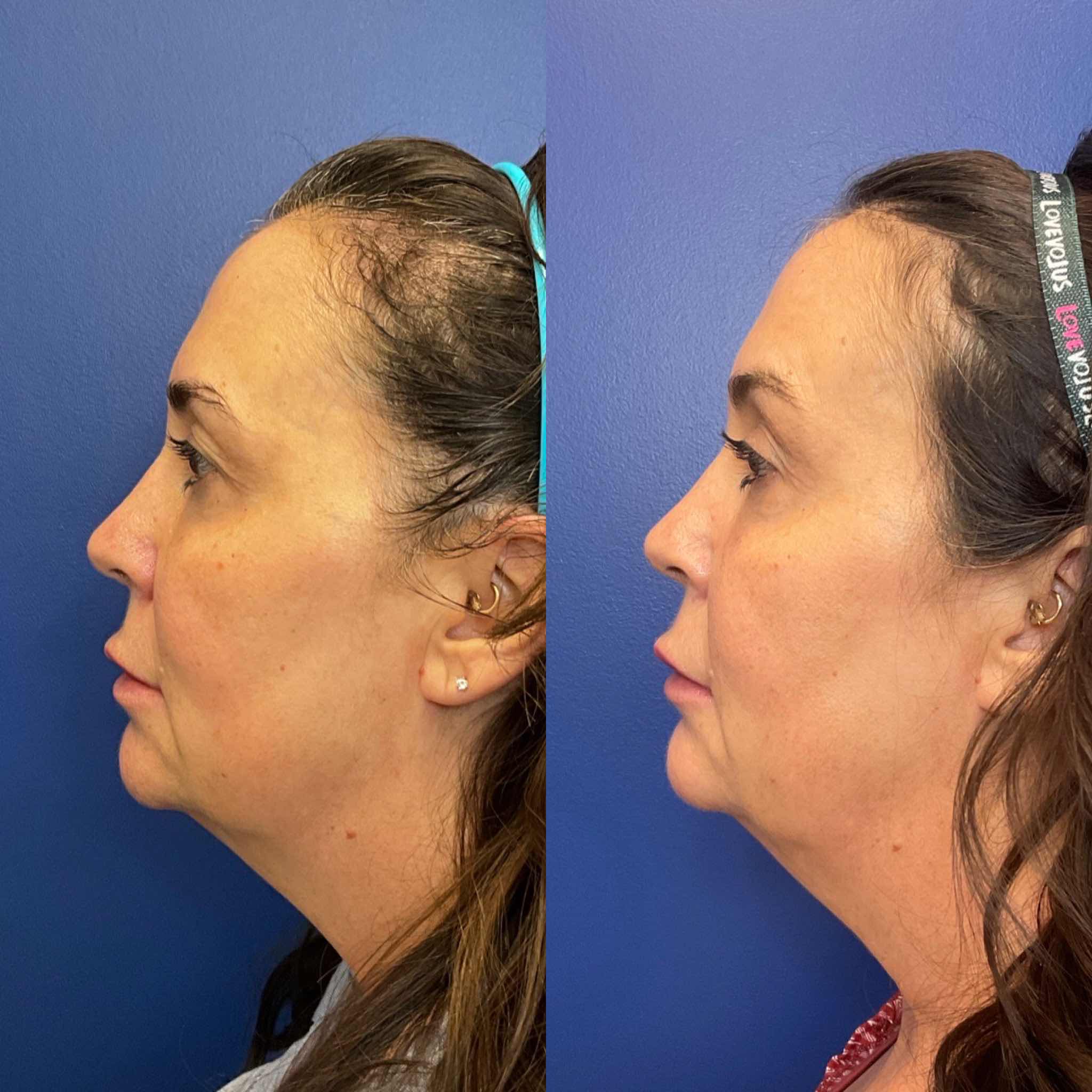
**

**Figure S13. Before and 6 months after hyperdilute CaHA-CMC during concurrent weight loss in a 53-year-old patient who lost approximately 16 pounds during the study.**

**
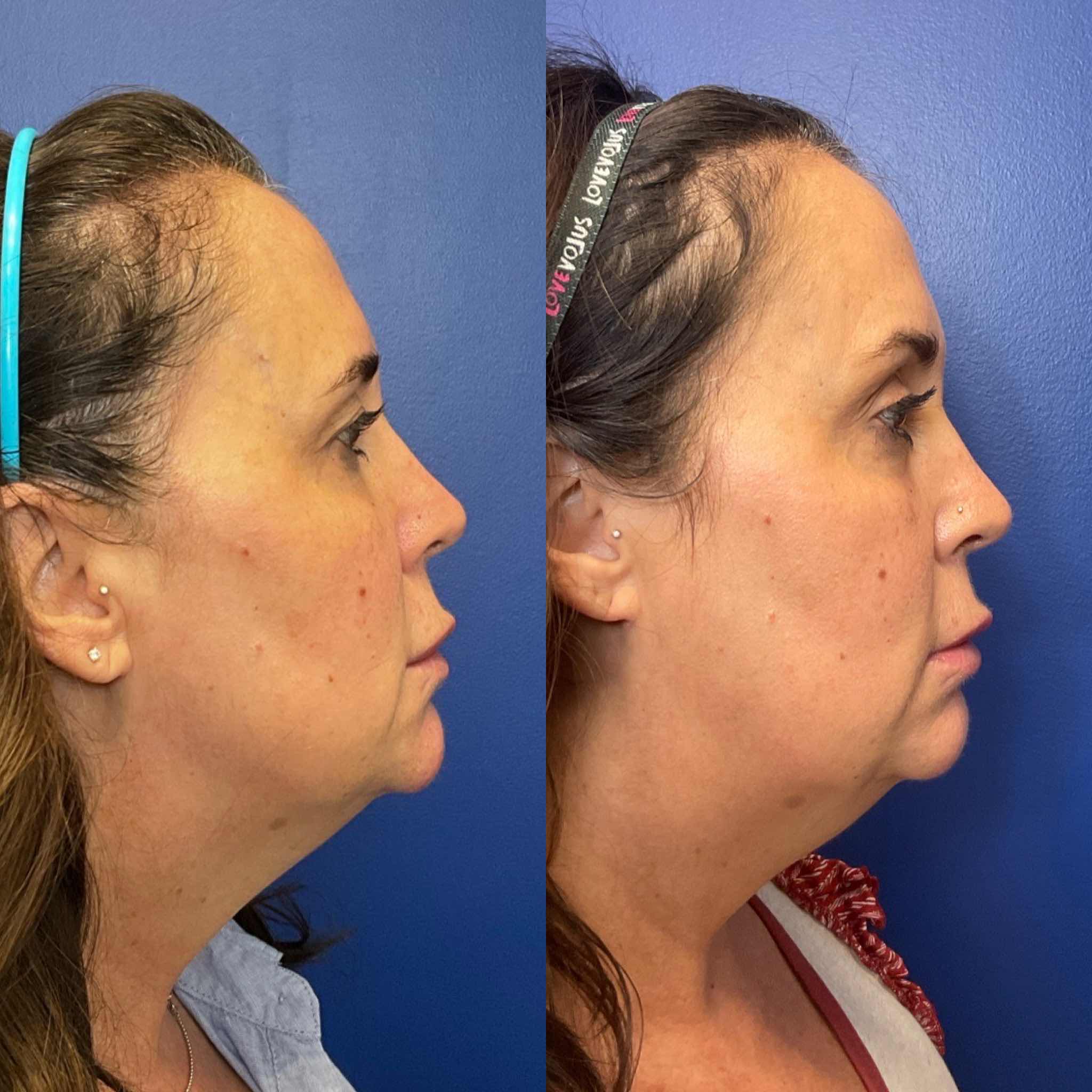
**

**Figure S14. Before and 6 months after hyperdilute CaHA-CMC during concurrent weight loss in a 53-year-old patient who lost approximately 16 pounds during the study.**

**
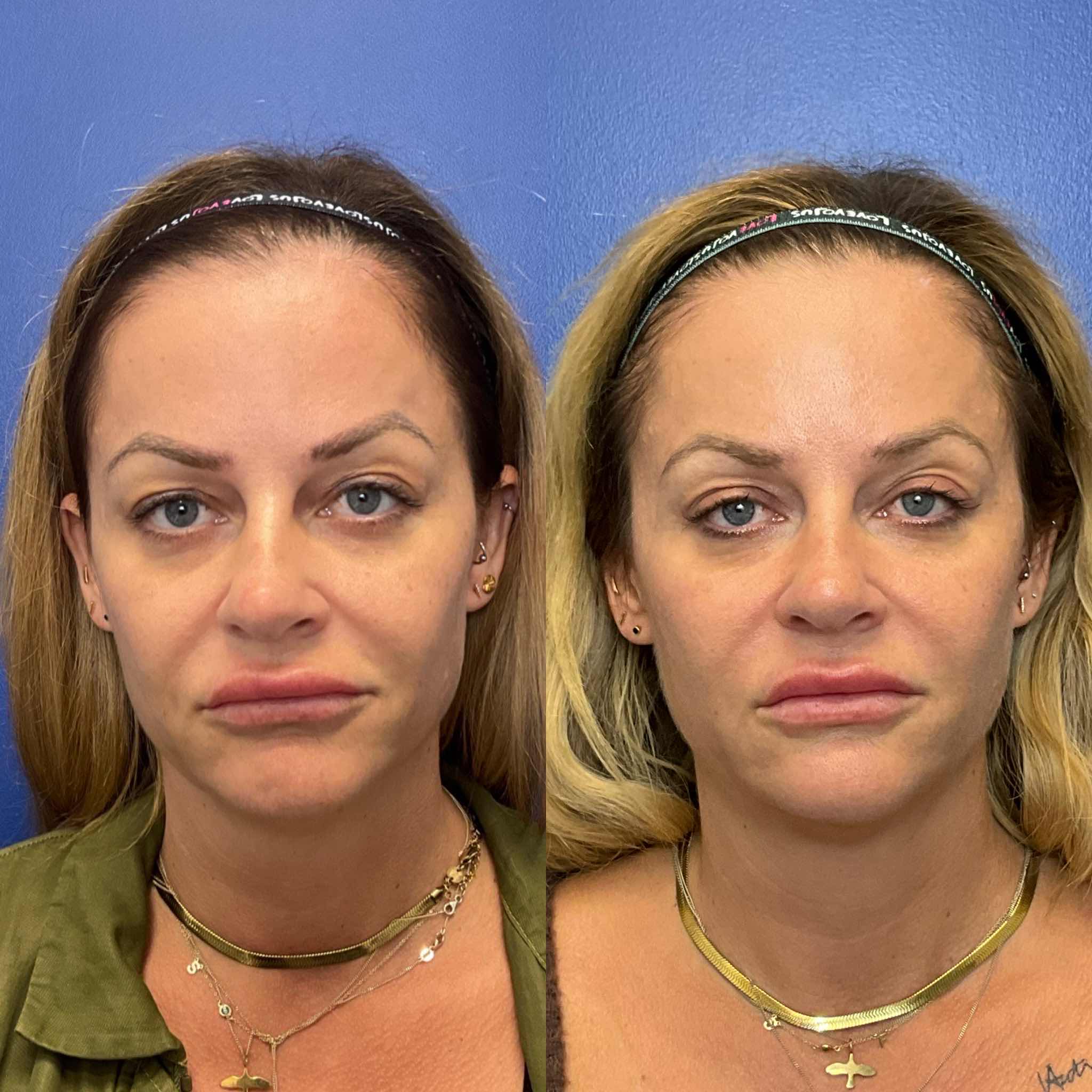
**

**Figure S15. Before and 6 months after hyperdilute CaHA-CMC during concurrent weight loss in a 45-year-old patient who lost approximately 34 pounds during the study.**

**
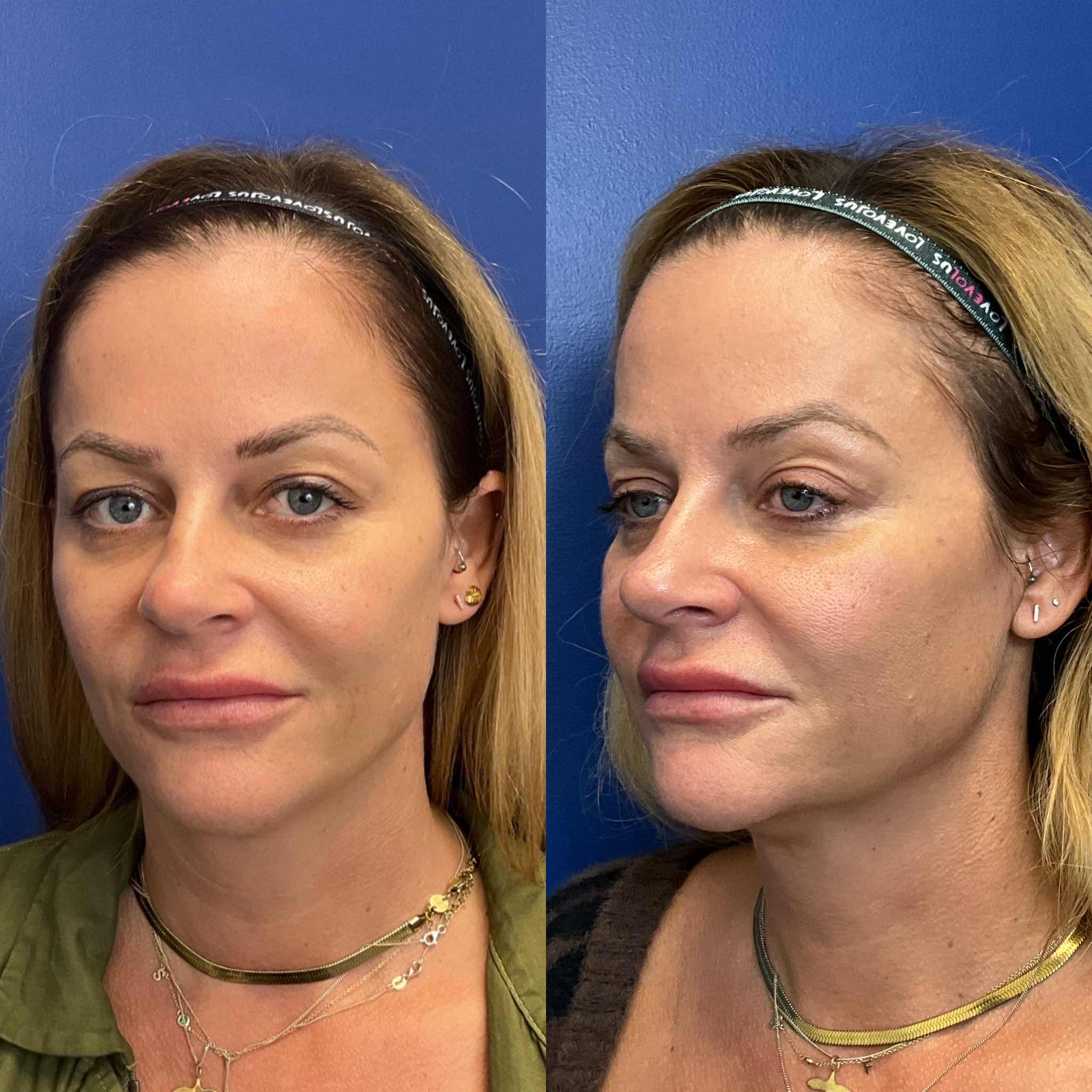
**

**Figure S16. Before and 6 months after hyperdilute CaHA-CMC during concurrent weight loss in a 45-year-old patient who lost approximately 34 pounds during the study.**

**
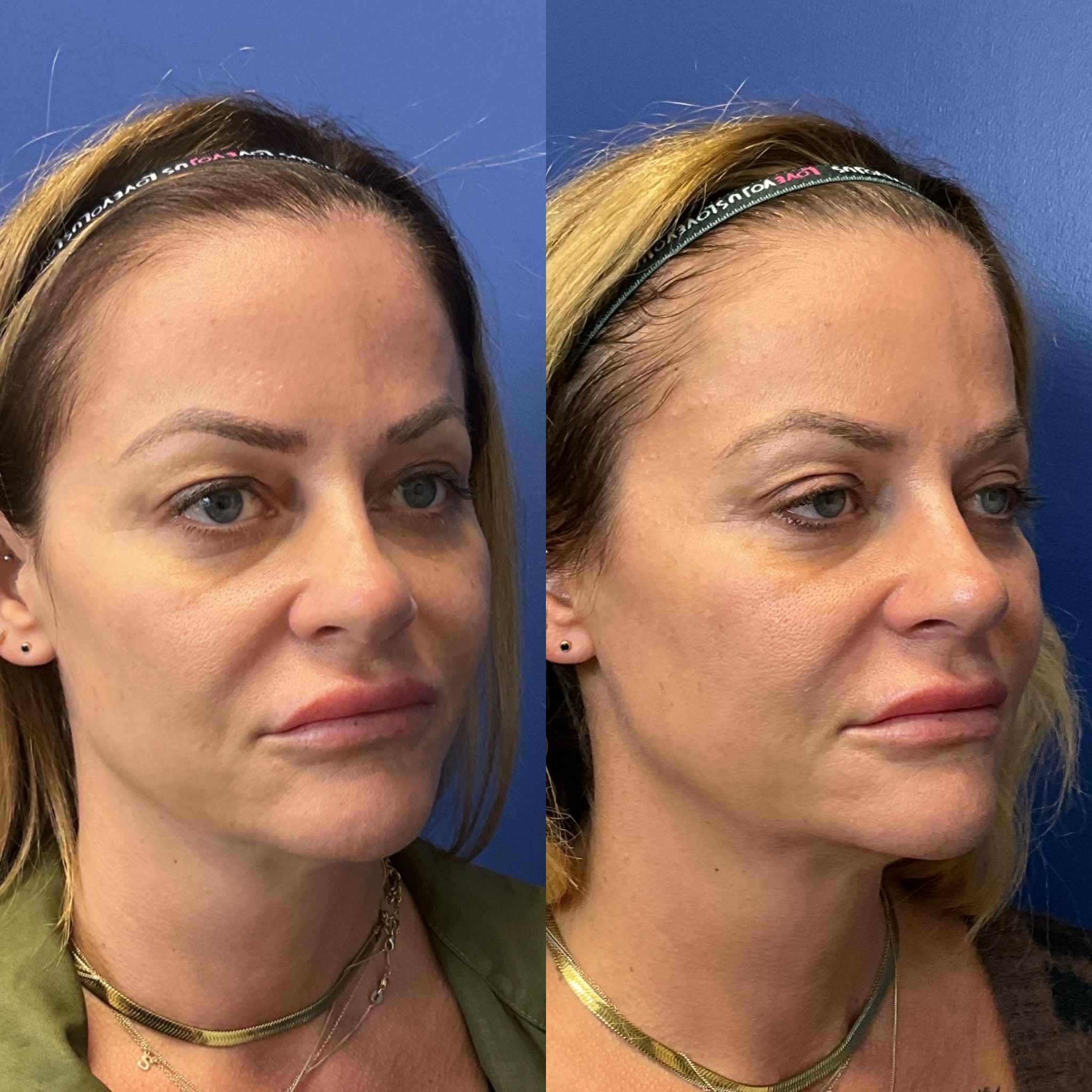
**

**Figure S17. Before and 6 months after hyperdilute CaHA-CMC during concurrent weight loss in a 45-year-old patient who lost approximately 34 pounds during the study.**

**
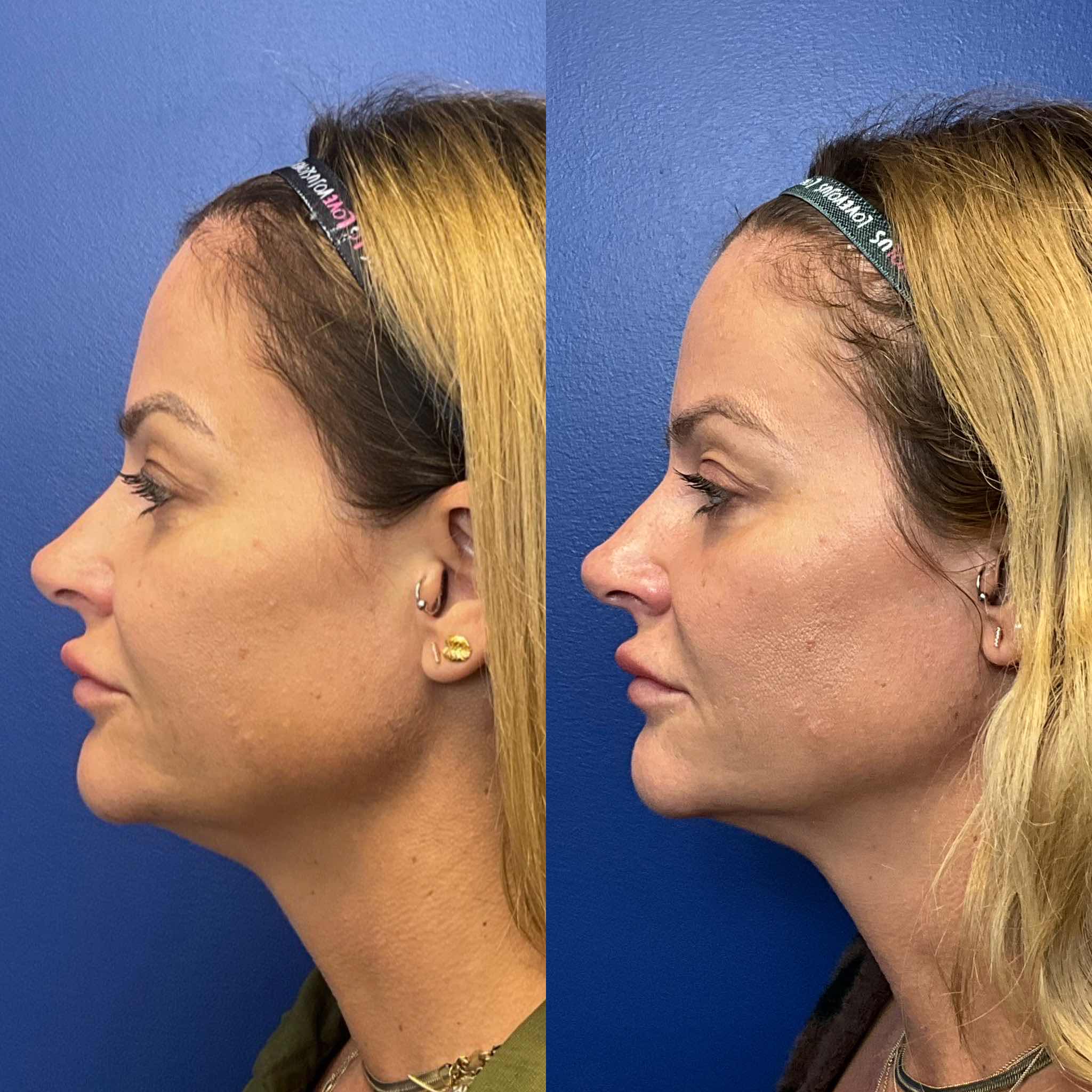
**

**Figure S18. Before and 6 months after hyperdilute CaHA-CMC during concurrent weight loss in a 45-year-old patient who lost approximately 34 pounds during the study.**

**
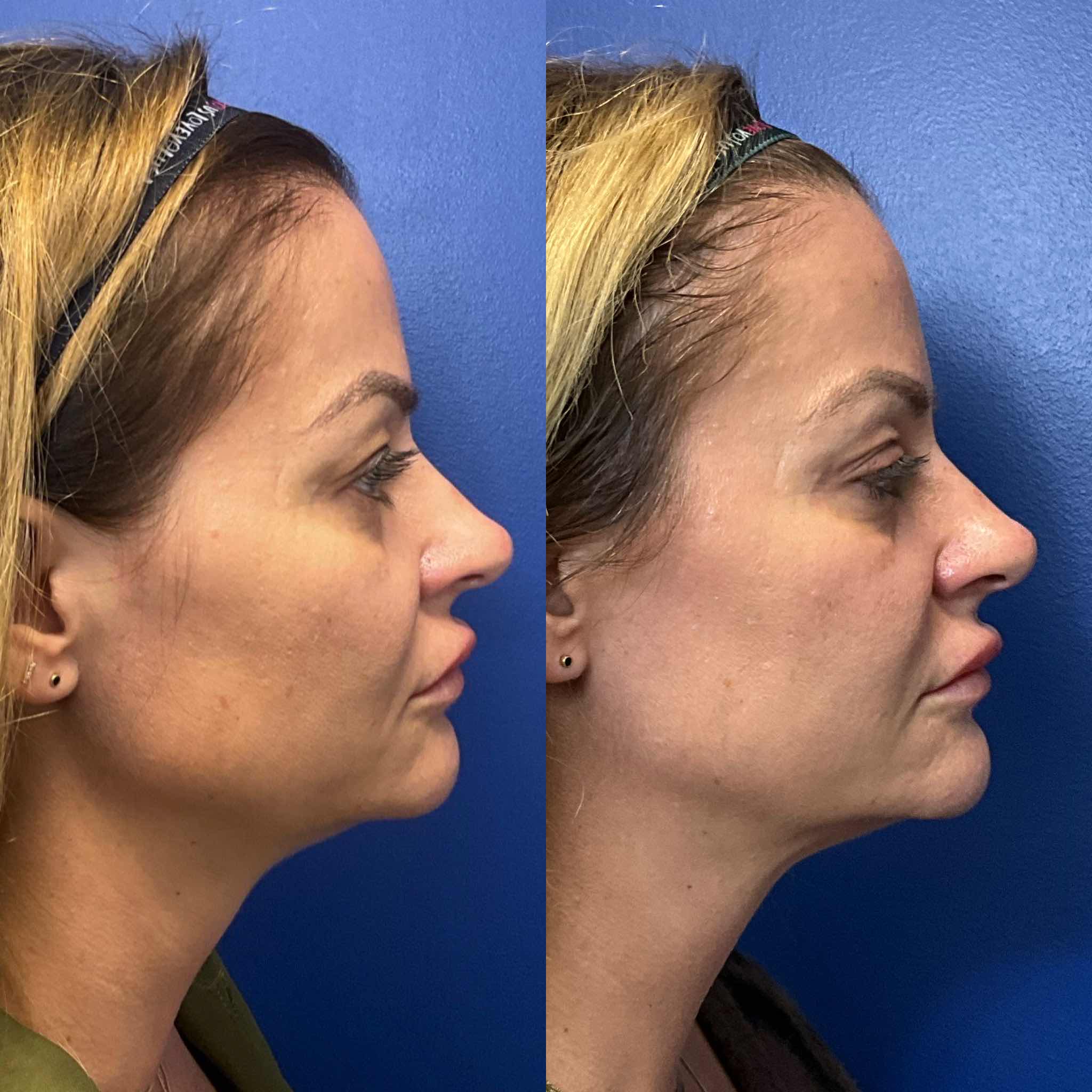
**

**Figure S19. Before and 6 months after hyperdilute CaHA-CMC during concurrent weight loss in a 45-year-old patient who lost approximately 34 pounds during the study.**

**
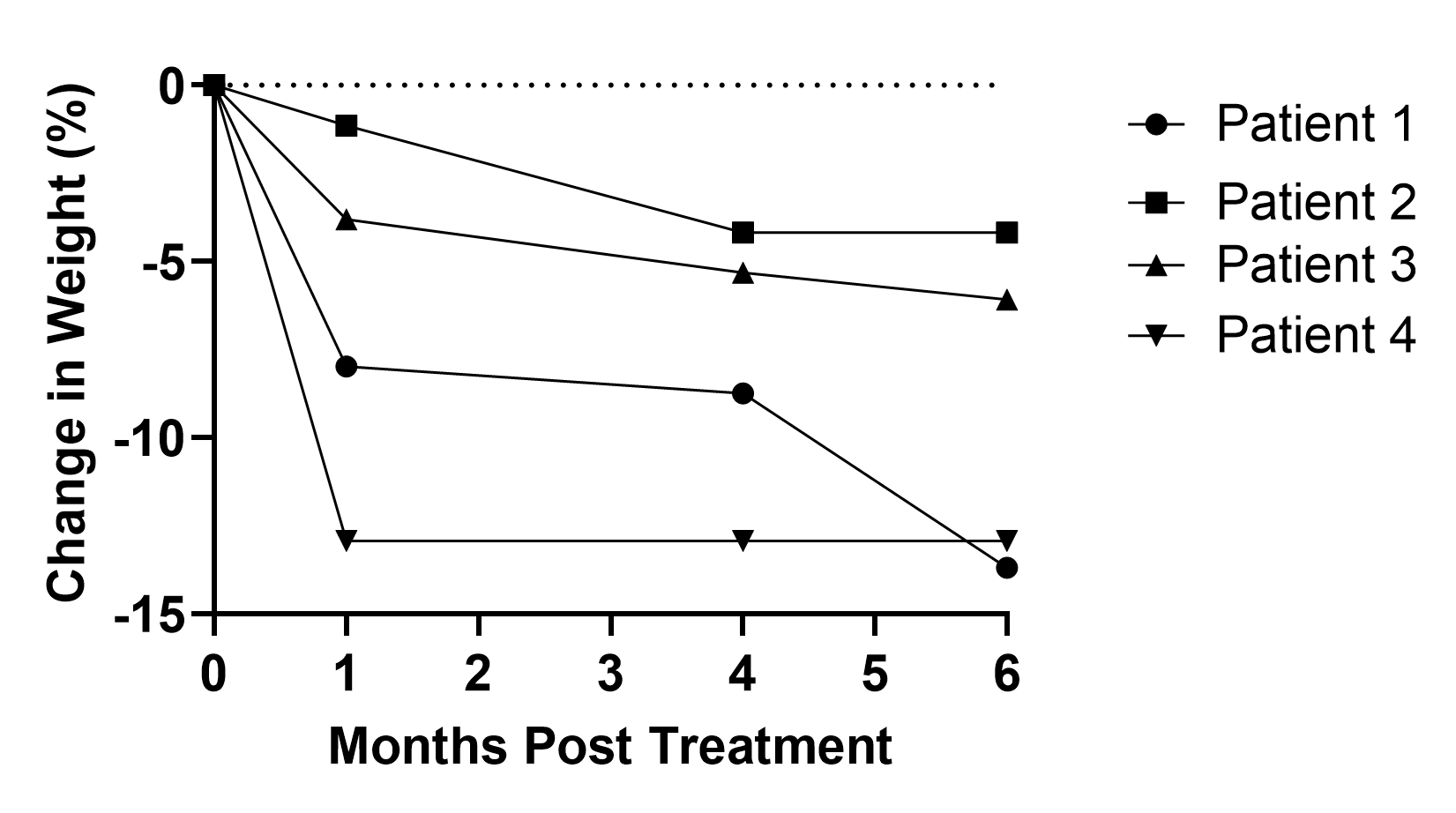
**

**Figure S20. Individual body weight loss for patients in this sub analysis.**

**
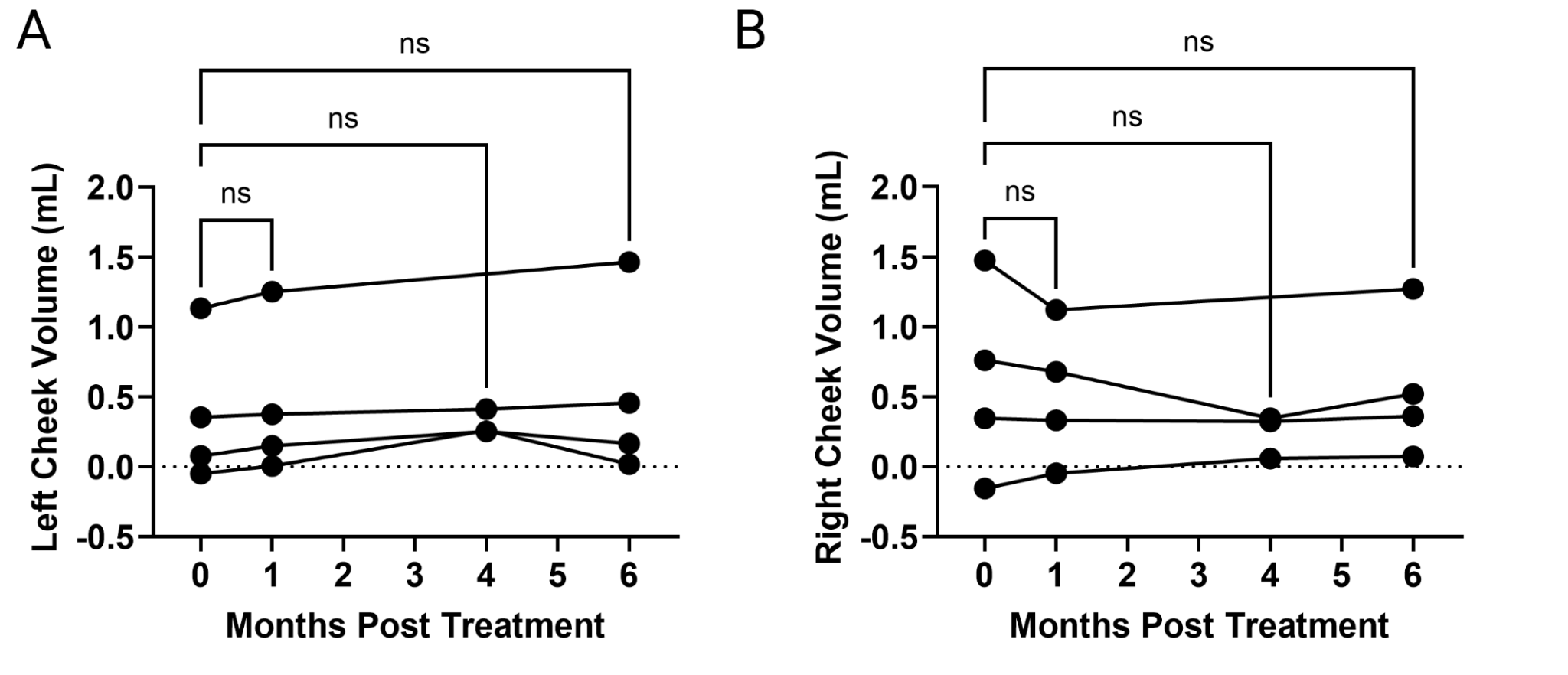
**

**Figure S21. (A) Left and (B) right cheek volumes measured in mL. No significant changes were observed across time points, indicating stable cheek volume despite rapid weight loss in GLP-1 agonist users. ns = not significant.**

**
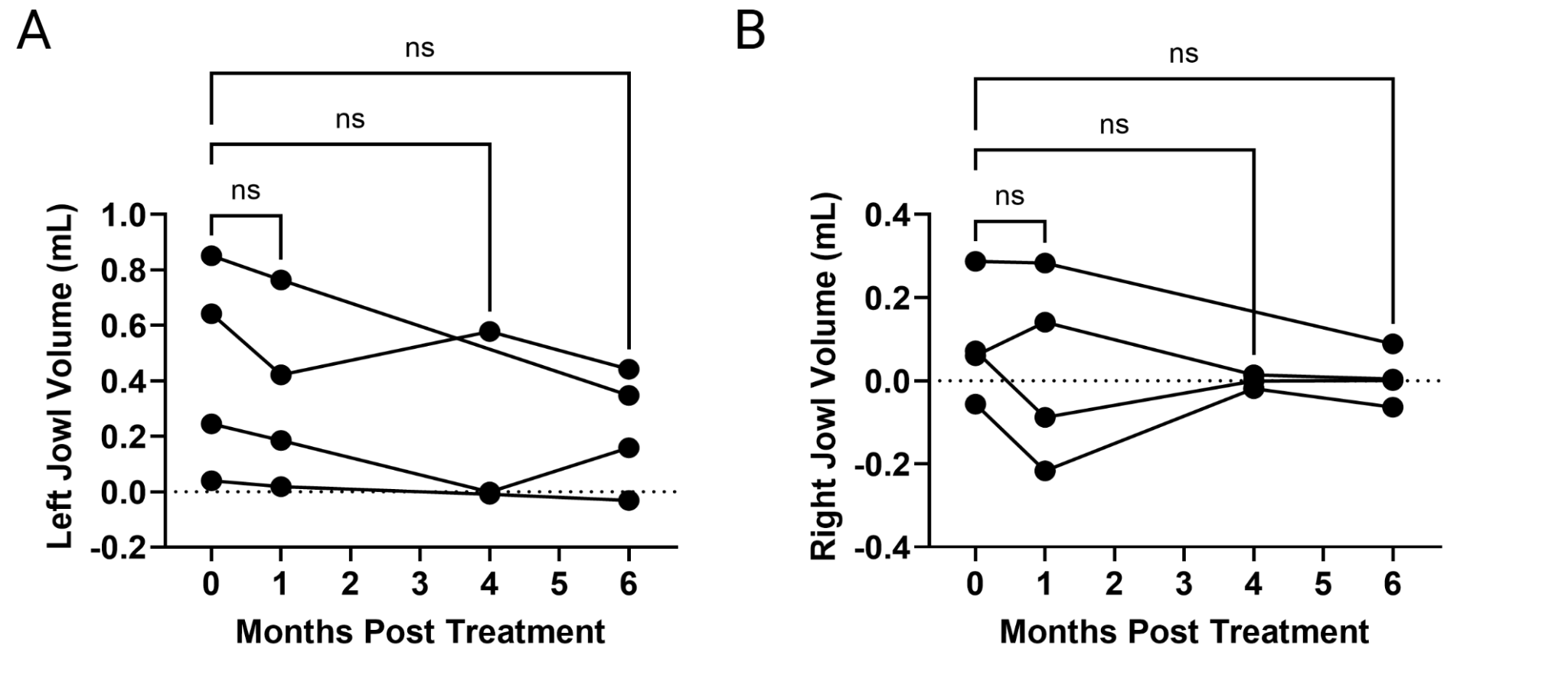
**

**Figure S22. (A) Left and (B) right jowl volumes measured in mL. No significant changes were observed across time points, indicating stable jowl volume despite rapid weight loss in GLP-1 agonist users. ns = not significant.**

**
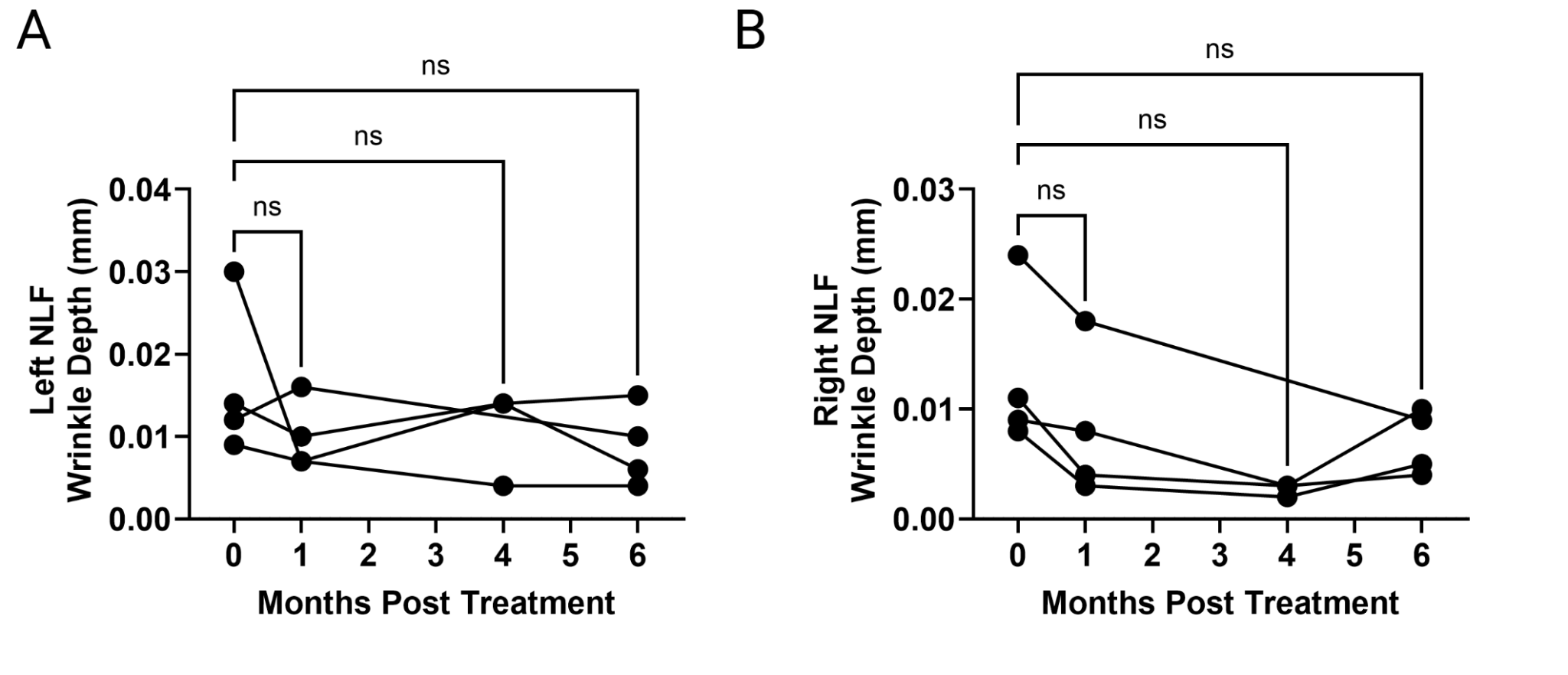
**

**Figure S23. (A) Left and (B) nasolabial fold depth measured in mm. No significant changes were observed across time points, indicating stable nasolabial fold depth despite rapid weight loss in GLP-1 agonist users. ns = not significant.**

**
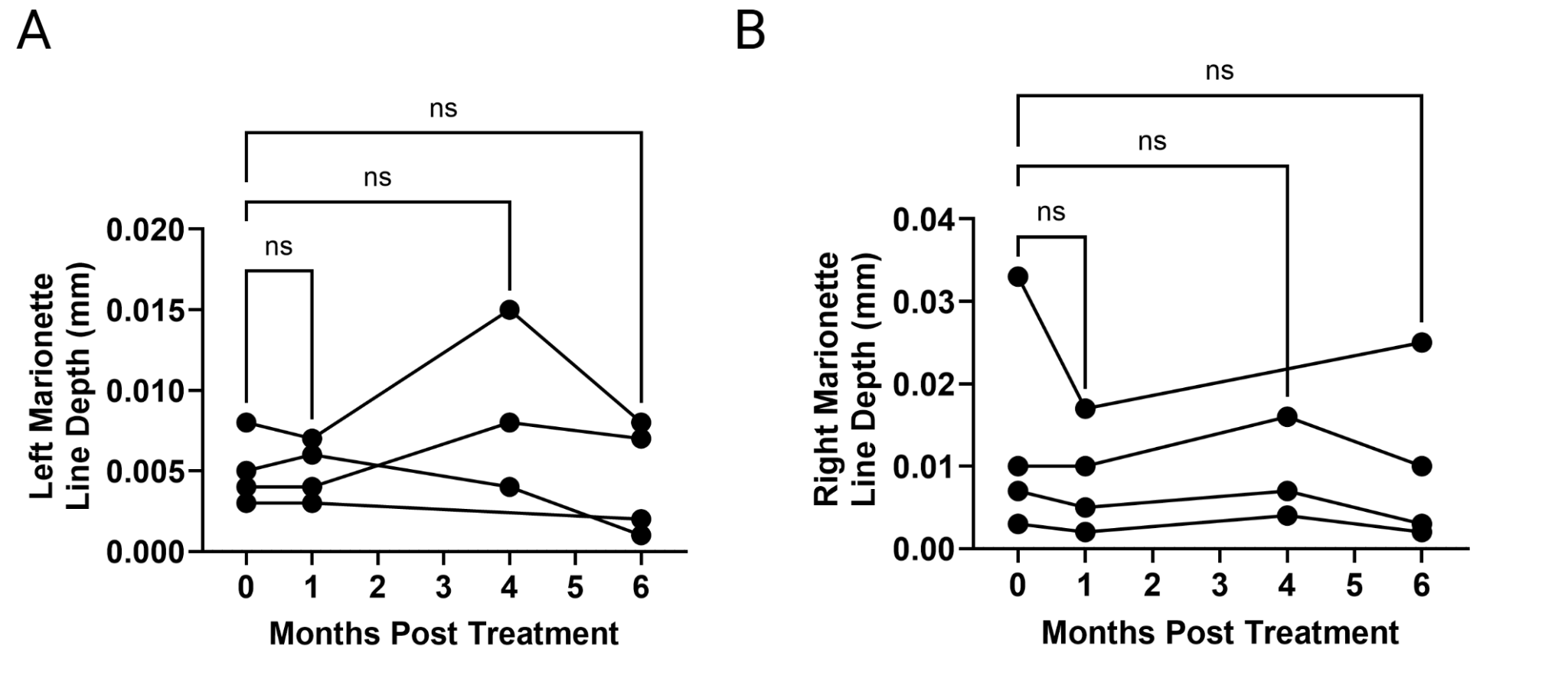
**

**Supplemental Figure 24. (A) Left and (B) right marionette wrinkle depth measured in mm. No significant changes were observed across time points, indicating stable marionette wrinkle severity despite rapid weight loss in GLP-1 agonist users. ns = not significant.**

**
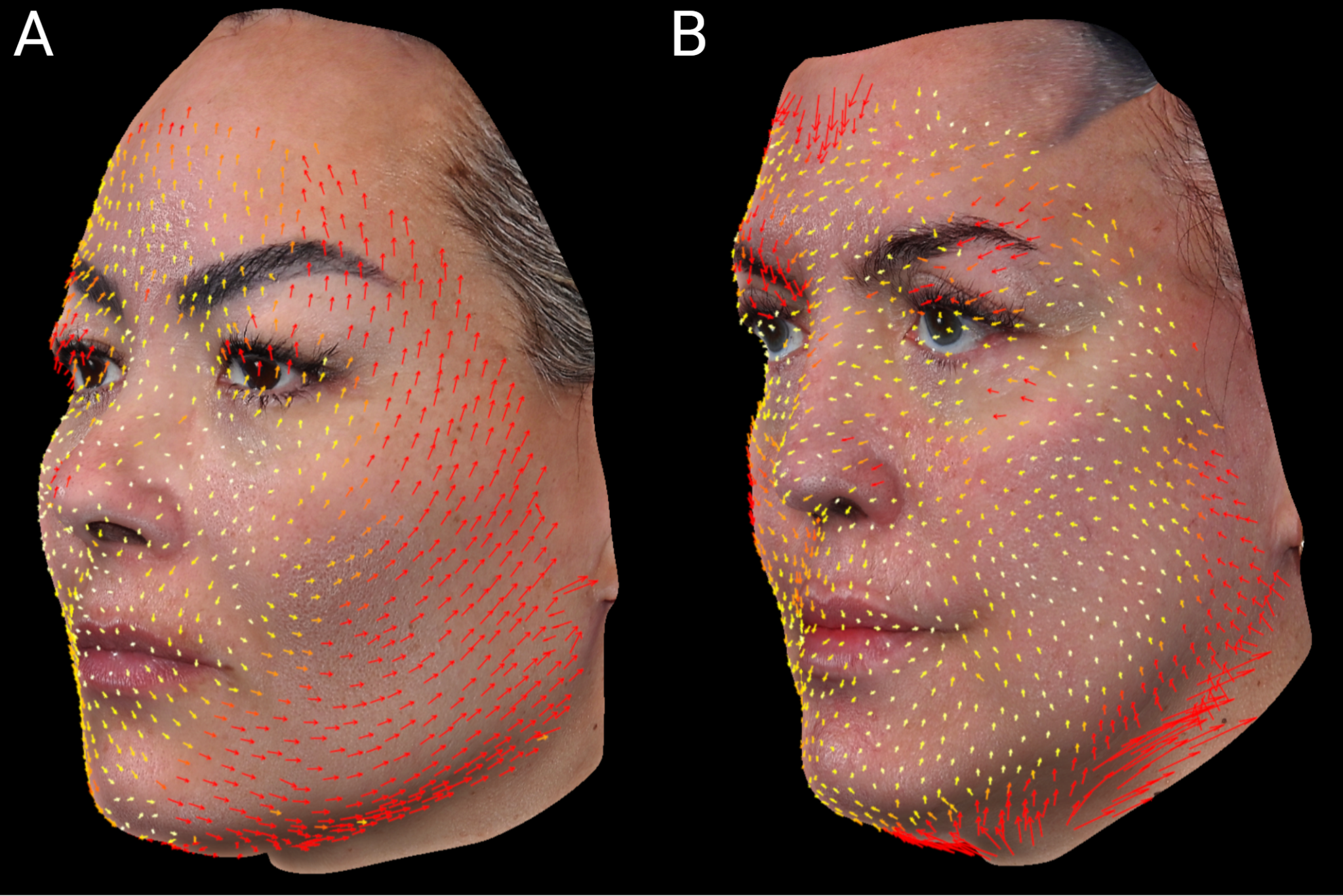
**

**Figure S25. Vector displacement analysis from baseline to 6 months post-treatment in female patients undergoing GLP-1RA-induced weight loss. (A) 49-year-old who lost 11 lb. (B) 54-year-old who lost 36 lb. Arrows represent soft-tissue movement; color indicates vector magnitude (red = greatest, yellow = moderate, pale yellow = minimal). In Panel A, superolateral vectors in the midface suggest active soft-tissue displacement, while in Panel B, shorter, more medial vectors may reflect positional preservation in the setting of greater weight loss. Upward vectors in the lower face in both patients indicate improved jowl contour and reduced skin laxity.**

**
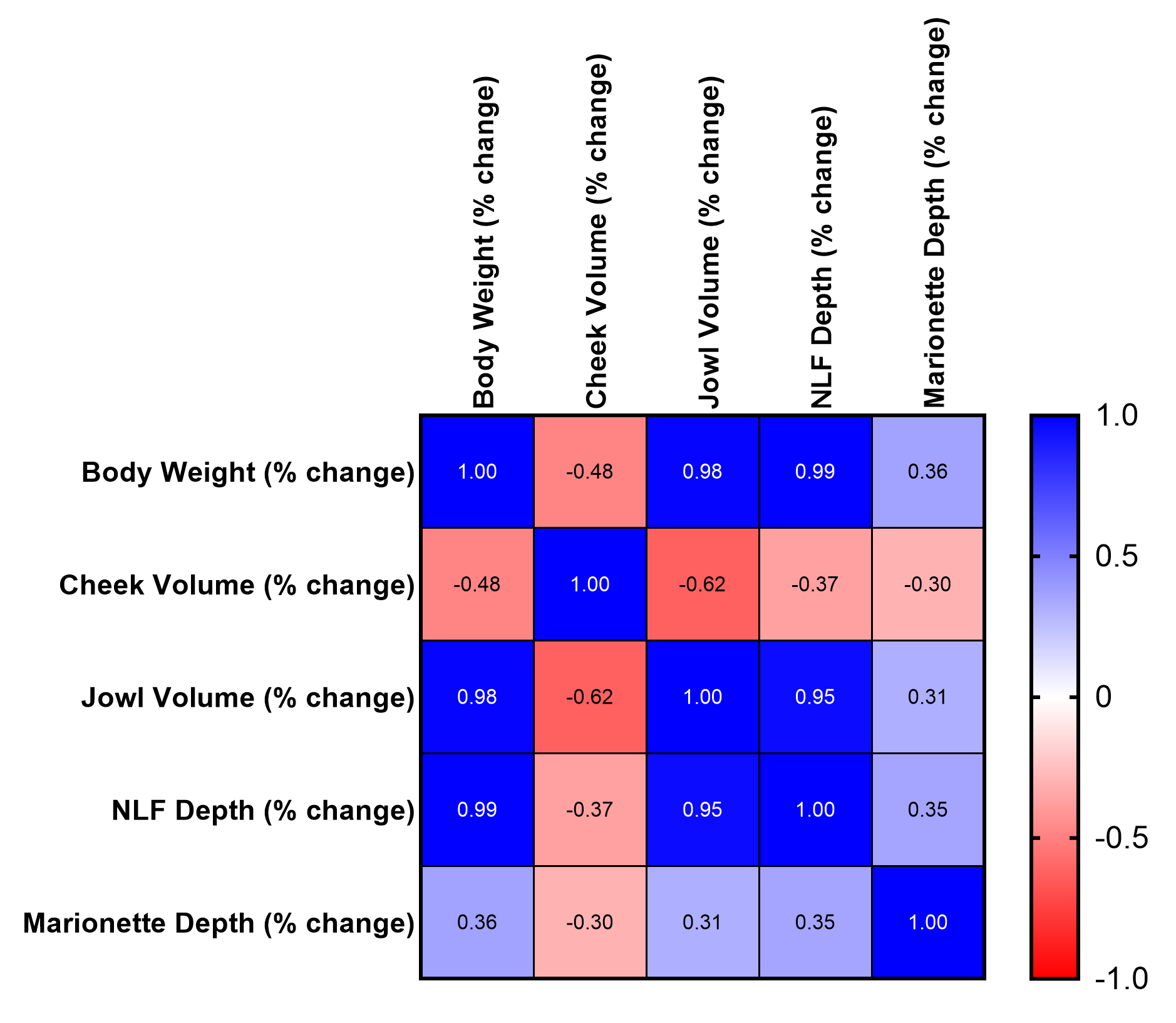
**

**Figure S26. Pearson’s r correlation matrix showing (red) negative r values and (blue) positive r values between correlates.**

**
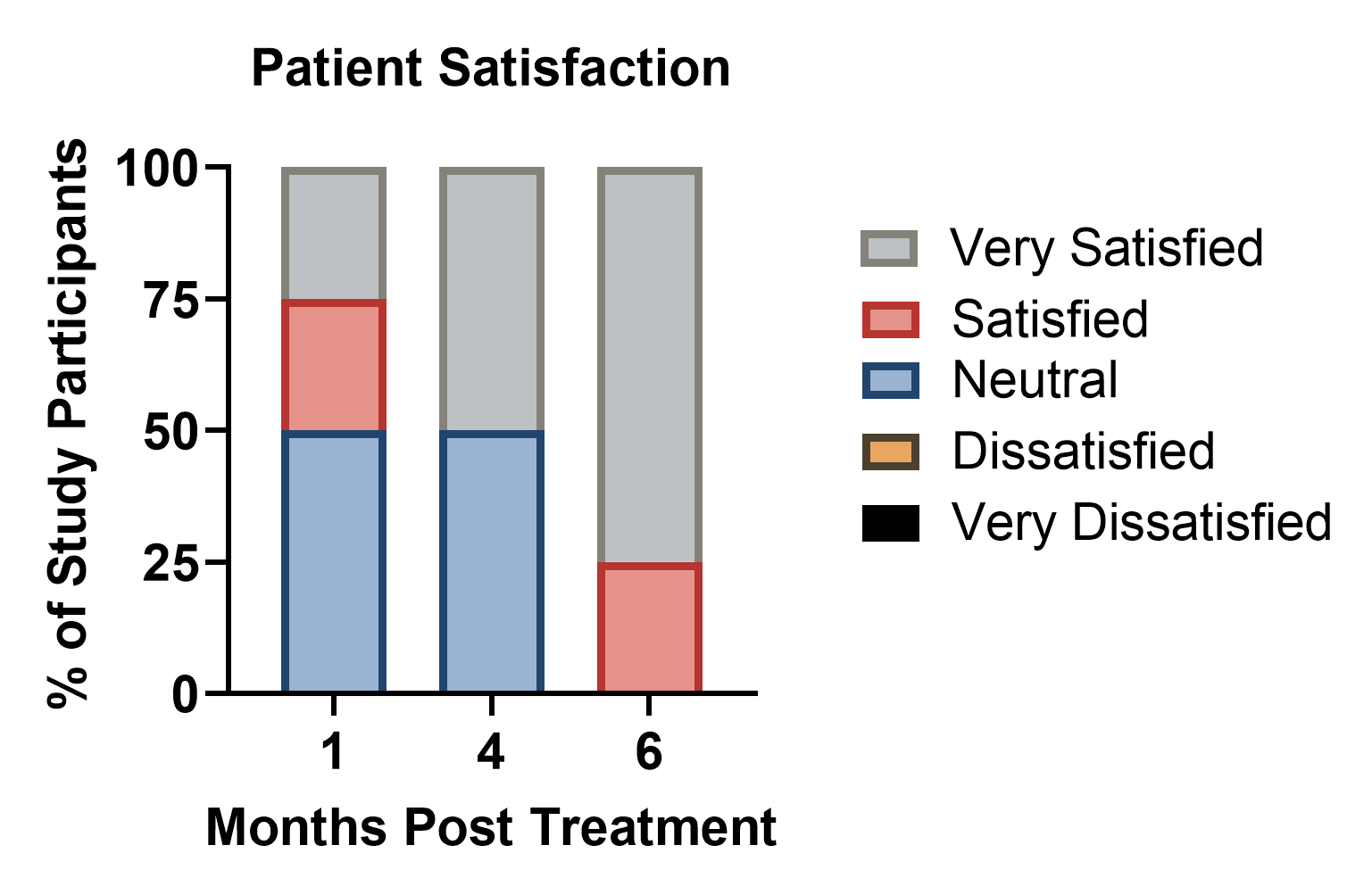
**

**Figure S27. Patient satisfaction measured on a 5-point scale 1-, 4-, and 6-months post-treatment.**
